# Styleworts under the microscope: a taxonomic account of *Levenhookia* (Stylidiaceae)

**DOI:** 10.3897/phytokeys.151.51909

**Published:** 2020-06-12

**Authors:** Juliet A. Wege

**Affiliations:** 1 Western Australian Herbarium, Biodiversity and Conservation Science, Department of Biodiversity, Conservation and Attractions, 17 Dick Perry Ave Kensington, Western Australia 6151, Perth, Australia Western Australian Herbarium Perth Australia

**Keywords:** Annual herbs, conservation, Flora of Australia, herbarium collections, taxonomy

## Abstract

A taxonomic revision of the Australian endemic genus *Levenhookia* R.Br. (Stylidiaceae) recognises 12 species, of which *L. aestiva* Wege, **sp. nov.** from south-western Australia is newly described. *Levenhookia preissii* (Sond.) F.Muell. is lectotypified and recircumscribed as a Swan Coastal Plain endemic, resulting in its addition to the *Threatened and Priority Flora List for Western Australia*. Lectotypes are also selected for *L. dubia* Sond., *L. leptantha* Benth., *L. sonderi* (F.Muell.) F.Muell. and *L. stipitata* (Benth.) F.Muell. ex Benth. Verification of herbarium records has expanded the known distribution of *L. murfetii* Lowrie & Conran and *L. pulcherrima* Carlquist and has confirmed the widespread distribution of *L. dubia* across southern Australia including Tasmania, where it is currently listed as extinct-surveys based on information gleaned from historical collections may lead to its rediscovery in this State. Descriptions, distribution maps and photographs for all species are provided along with a key to species.

## Introduction

**Figure 1.**
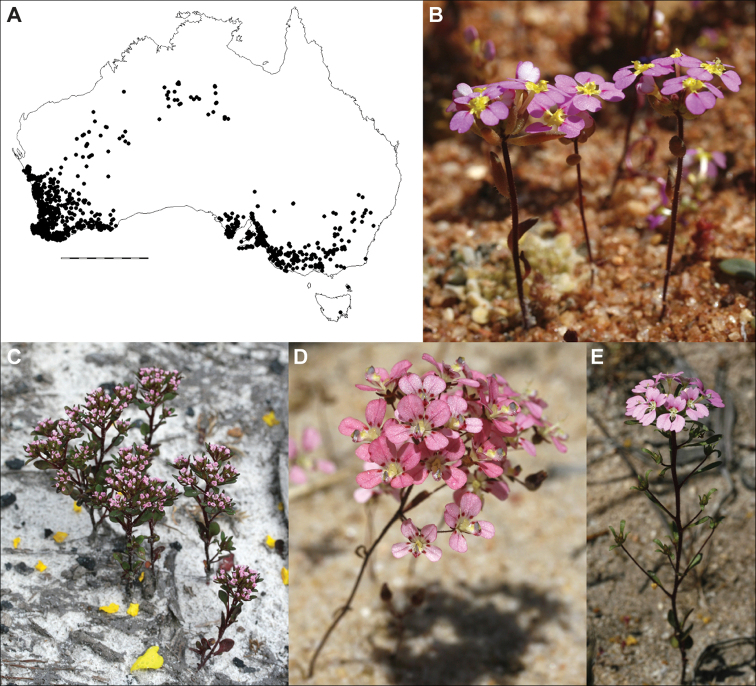
*Levenhookia* is a genus of annual herbs endemic to Australia **A** distribution in temperate and semi-arid regions of Australia, scale bar 1000 km **B***L. leptantha* (*J.A. Wege 2063*) **C***L. pusilla* (*J.A. Wege 1749 & W.S. Armbruster*) **D***L. octomaculata* (*J.A. Wege 2074*) **E***L. pulcherrima* (*J.A. Wege 1937*). Photos by J.A. Wege.

The Stylewort genus *Levenhookia* R.Br. (Stylidiaceae: Asterales) is endemic to the southern temperate and arid zones of Australia and comprises 12 mostly diminutive annual species (Fig. 1) with delicate and intricate flowers that are best observed under magnification. The genus was established by Robert Brown (1810) who aptly named it for Antony van Leeuwenhoek (1632–1723), a Dutch shopkeeper and celebrated microscopist who crafted optical lenses and bespoke microscopes and who made an extraordinary series of novel microscopic findings, including on protists, bacteria, spermatozoa and rotifers (Miall 1912; Dobell 1932). Although readily overlooked due to their size, species of *Levenhookia* can be impressive en masse, often growing in localised colonies of hundreds or thousands of plants. They are found in a wide variety of habitats, especially in south-western Australia where all but two of the species occur.

**Figure 2.**
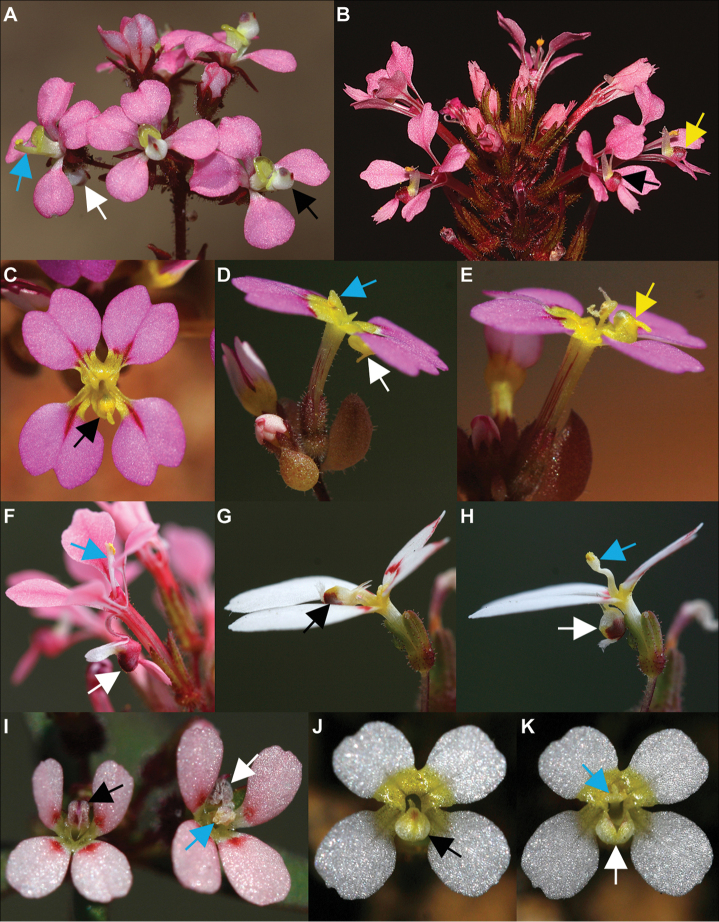
Labellum and column movement in *Levenhookia*. Black arrow = labellum hooded over column; white arrow = labellum ‘triggered’ to release column; yellow arrow = labellum repositioned following column release; blue arrow = column position immediately following release from labellum **A***L. stipitata* (unvouchered, Augusta area, Western Australia) **B***L. aestiva* (*J.A. Wege 2090*) **C–E***L. leptantha* (*J.A. Wege 2063*), with labellum enclosing the column (C), triggered to release the column which moves to the opposite side of the flower (D) and subsequently repositioned (E) with the stigmatic lobes developed **F***L. aestiva* (*J.A. Wege 2090*), labellum triggered with column momentum stopped by the sheath at the base of the corolla lobes **G, H***L. pauciflora* (at *J.A. Wege 1071 & C. Wilkins*): note the unusual, distally-angled column and brush-tipped labellum **I***L. murfetii* (*J.A. Wege 2060*): note the dorsal position of the labellum; **J, K***L. dubia*, with the labellum opening (but not otherwise moving) to release the column (**K**). Photos by R.W. Davis (**A, B**) and J.A. Wege (**C–K**).

*Levenhookia* is the second largest genus in Stylidiaceae, a family dominated by the triggerplants (*Stylidium* Sw. ex Willd.), a diverse genus (> 300 spp.) renowned for its protandrous flowers with a touch-sensitive floral column that rapidly places pollen on, or retrieves pollen from, visiting insects (Erickson 1958; Findlay and Findlay 1975; Armbruster et al. 1994). Both genera have zygomorphic flowers with five corolla lobes, one of which (the labellum) is usually highly modified and variously ornate. In *Stylidium*, the labellum is typically reflexed and reduced in size to accommodate the column movement. In contrast, the labellum in *Levenhookia* is uniquely hooded (Fig. 2), enclosing the distal end of the column where the reproductive organs are located, and touch-sensitive (Brown 1810; Sargent 1918; Erickson 1958), springing backwards or opening when stimulated to expose the column (which can exhibit limited movement as a result). Unlike *Stylidium*, in which the column resets and retriggers after a short period of time, the movement of the labellum in *Levenhookia* and concomitant release of the column occurs just once.

Research by Brown (1810), Bentham (1837, 1868), Sonder (1845) and Mueller (1855, 1862, 1867) cumulatively led to the discovery of seven species of *Levenhookia*, three of which were originally named in the novel genus *Coleostylis* Sond. (Sonder 1845; Mueller 1855), but later transferred to *Levenhookia* (Mueller 1858, 1864). Although a further four species have since been described (Erickson and Willis 1956, 1965; Carlquist 1969; Lowrie and Conran 2011), the genus lacks a modern taxonomic account, the most recent being that of Mildbraed (1908). That said, knowledge of the genus was greatly advanced by Erickson (1958) who observed a range of species in the wild and, like Leeuwenhoek, had a keen eye for detail.

Underpinning the present taxonomic study are 1740 collections of *Levenhookia* housed at various Australian herbaria, ca. 75% of which have been collected since the publication of Erickson’s (1958) popular account. Sorting and verifying this material has been an arduous task given the size of the plants and the limited number of individuals associated with some collections; however, it has led to a much improved understanding of the distribution and conservation status of the included taxa. Herbarium-based studies also precipitated the discovery of a new species (*L. aestiva* Wege sp. nov.) endemic to the far south-west of Australia, material of which had previously been included under a broad concept of *L. preissii* Sond. (Sonder 1845; Mildbraed 1908; Erickson 1958; Wheeler 1987, 2002; Lowrie and Conran 2011). *Levenhookia preissii* is now considered to have a more restricted distribution on the heavily cleared landscape of Western Australia’s Swan Coastal Plain bioregion and has recently been conservation-listed (Western Australian Herbarium 1998–). In contrast, the geographic distributions of *L. pulcherrima* Carlquist and *L. murfetii* Lowrie & Conran have been expanded through verification of herbarium records, the latter by more than 600 km (although the former remains poorly known).

Examination of historical collections has also proven invaluable, notably in the case of *L. dubia* Sond., a species widespread across southern Australia, but listed as extinct in Tasmania (with some uncertainty as to whether it ever occurred there: see Gray 2011). Specimens from Flinders Island and Pontville that were collected in the mid- to late 1800s, have been located at the National Herbarium of Victoria and not only confirm that it was indeed present in Tasmania, but can be used to provide information for future survey effort. Consideration of historical type material has also revealed several typification issues that are resolved herein to deliver a robust taxonomic framework.

## Methods

This study is primarily based on the examination of herbarium material and associated spirit and photographic collections at the Western Australian Herbarium (PERTH), South Australian Herbarium (AD), Australian National Herbarium (CANB), Northern Territory Herbarium (DNA) and National Herbarium of Victoria (MEL). Specimens at the National Herbarium of New South Wales (NSW) were unavailable for study during this project. Distribution statements and maps are based on taxonomically validated specimen data, though collections of *L. dubia* from New South Wales that are housed at NSW have been included (these are likely to be correctly identified since this is the only species of *Levenhookia* recorded in this State). Type specimens at a range of additional institutions were also examined during research visitations, via specimen loans or through digital portals (indicated by image!): acronyms follow Thiers (2019). Geographic regions follow the *Interim Biogeographic Regionalisation for Australia* (Department of the Environment 2013).

Vegetative characters were mostly coded from pressed specimens, whereas floral characters were largely scored from wet collections, but supplemented with information gleaned from pressed material, field observations (mostly in south-western Australia) and photographic records. The exception is *L. preissii*, which has been described solely from pressed material and associated images, with flowers reconstituted from *G. Woodman Opp 5* (PERTH) and *B.J. Keighery 2546* (PERTH). Spirit collections from the following populations were consulted: *L. aestiva* – *J.A. Wege 1590* (PERTH), *J.A. Wege 1592* (PERTH), *J.A. Wege 2090* (PERTH); *L. chippendalei* F.L.Erickson & J.H.Willis – *K. Coate 372* (PERTH), *K.F. Kenneally & D.J. Edinger K 12671 E 3868* (PERTH); *L. dubia* – *H.S. Meyer 23* (MEL), *T.B. Muir 6742* (MEL), *J.A. Wege 376*, *R. Butcher & C. Wilkins* (PERTH), *J.A. Wege 1773 & C. Wilkins* (PERTH), *J.A. Wege 1836 & B.P. Miller* (PERTH), *J.A. Wege 2062* (PERTH); *L. leptantha* – *J.A. Wege 251 & N. Siemon* (PERTH), *J.A. Wege 895 & C. Wilkins* (PERTH), *J.A. Wege 1828 & K.R. Thiele* (PERTH), *J.A. Wege 2063* (PERTH); *L. murfetii* – *J.A. Wege 360*, *R. Butcher & C. Wilkins* (PERTH), *J.A. Wege 1829 & K.R. Thiele* (PERTH), *J.A. Wege 2060* (PERTH), *J.A. Wege 2066* (PERTH), *J.A. Wege 2067* (PERTH); *L. octomaculata* F.L.Erickson & J.H.Willis – *J.A. Wege 2074* (PERTH); *L. pauciflora* Benth. – *J.A. Wege 209 & L. Cobb* (PERTH), *J.A. Wege 380*, *R. Butcher & C. Wilkins* (PERTH), *J.A. Wege 1071 & C. Wilkins* (PERTH); *L. pulcherrima* – *J.A. Wege 1937* (PERTH); *L. pusilla* – *J.G. Eichler s.n.* (MEL 1580366), *A.S. George 11712* (PERTH), *K.A. Shepherd 1139 & J.A. Wege* (PERTH), *J.A. Wege 795* (PERTH), *J.A. Wege 1051 & C. Wilkins* (PERTH), *J.A. Wege 1749 & W.S. Armbruster* (PERTH); *L. sonderi* (F.Muell.) F.Muell. – *D.E. Murfet 3317* (AD); *L. stipitata* – *J.A. Wege 120* (PERTH), *J.A. Wege 156 & P. French* (PERTH), *J.A. Wege* 777 (PERTH), *J.A. Wege 1562 & B.P. Miller* (PERTH), *J.A. Wege 1688 & W.S. Armbruster* (PERTH), *J.A. Wege 1873* (PERTH), *J.A. Wege 1874* (PERTH).

## Taxonomic treatment

### Levenhookia

Taxon classificationPlantaeAsteralesStylidiaceae

R.Br., Prodr. Fl. Nov. Holland.: 572. 1810

3F017EA0-C9A5-52A5-B3A2-E66C2578D7C4

Leeuwenhoekia , orth. var.: C.P.J. Sprengel, Anleit. Kenntn. Gew. ed. 2, 2(1): 300. 1818.
Levenhoekia , orth. var.: E.G. Steudel, Nom. Bot. 1: 477. 1821.
Leeuwenhookia , orth. var.: H.G.L. Reichenbach, Consp. Regn. Veg.: 91. 1828.
Leuwenhoekia , orth. var.: F.G. Bartling, Ord. Nat. Pl. 149. 1830.
Leeuwenhockia , orth. var.: E.G. Steudel, Nom. Bot., edn 2(2): 21. 1841.
Leeuvenhookia , orth. var.: F.J.H. von Mueller, Fragm. 1(1): 18. 1858.
Leewenhoekia , orth. var.: F.J.H. von Mueller, Syst. Census Austral. Pl: 86. 1882.
Coleostylis Sond. in J.G.C. Lehmann, Pl. Preiss. 1(3): 391. 1845. Lectotype, here designated: Coleostylis preissii Sond. [= Levenhookia preissii (Sond.) F.Muell.]
Coleostyles , orth. var.: G. Bentham & J.D. Hooker, Gen. Plant. 2(2): 535. 1876.

#### Type.

*Levenhookia pusilla* R.Br.

#### Description.

Diminutive annual herbs with simple or branched stems, usually glandular-hairy. Leaves scattered (rarely basally clustered), usually petiolate, margins entire. Inflorescence racemose, often corymbose or umbellate, sometimes reduced to a solitary flower, with a bract subtending each flower. Flowers bisexual, sometimes protandrous, zygomorphic. Calyx lobes 5, free (rarely with the anterior pair basally connate), margins entire. Corolla lobes 5, connate into a short to long tube with 4 spreading or distally recurved lobes and a smaller, highly modified labellum; labellum ventral or sometimes dorsal (rarely lateral) through rotation of the pedicel, galeiform (hooded), often ornate, covering the distal portion of the column, springing backwards or opening when stimulated to release the column; corolla outgrowths forming a column sheath at the throat, often nectariferous. Column immobile or with restricted movement upon release from the labellum; anthers 2, bilocular, pollen yellow; stigma 2-lobed. Ovary inferior, with many ovules on a free-central placenta. Nectary sometimes present on top of the ovary. Capsules dehiscent. Seeds small, brown, rugulose, sometimes papillate.

#### Number of species, distribution and ecology.

A genus of 12 species from a variety of habitats in southern temperate Australia, extending into the arid zone in Western Australia and the Northern Territory (Fig. 1A). The only representative of the genus in Tasmania is currently listed as extinct in that State (refer to the additional information provided under *L. dubia*). Some species are disturbance opportunists that are more readily found the year following a fire.

#### Floral morphology and pollination.

The hooded labellum is responsive to touch, usually springing backwards through rapid recurvature of its claw, releasing the column (Fig. 2A, D, F, H, I) before gradually repositioning itself at or above the level of the corolla lobes (Fig. 2B, E); it does not enclose the column or respond to stimuli again. *Levenhookia dubia* is an exception since its sessile labellum opens to release the column, but does not otherwise move (Fig. 2J, K). As first noted by Erickson (1958), the exact point of stimulus varies amongst species: probing the apex of the hood or apical appendage will trigger the release of the column in *L. dubia*, *L. leptantha*, *L. murfetii*, *L. octomaculata*, *L. pusilla* and *L. stipitata*, whereas the labellum in *L. aestiva* and *L. pauciflora* is more reliably stimulated by touching the basal appendages. Erickson (1958) observed the sensitive point to be in the throat of the flower in *L. preissii*, something which I intermittently observed in *L. aestiva*. Sometimes the labellum cannot be manually triggered and it has been hypothesised that flowers await a certain stage of maturity before becoming responsive (Sargent 1918; Erickson 1958). In the absence of an external stimulus, growth of the upper-most stigmatic lobe, or apparently of the column itself, can sometimes trigger labellum movement (Erickson 1958).

The action of the labellum usually results in a degree of column movement (Fig. 2D, F, H, K), although movement is slight or lacking in both *L. murfetii* (Fig. 2I) and *L. pusilla*. These small-flowered species appear to be autogamous: the anthers dehisce within the labellum and the stigmatic lobes develop concurrently, becoming covered in pollen (Sargent 1918; pers. obsv.). In *L. leptantha*, *L. aestiva* and *L. dubia*, the column moves from the anterior to the posterior side of the flower, coming to rest against the column sheath at the base of the corolla lobes (Fig. 2D, F, K, respectively) before gradually shifting to an erect or slightly forward-arched position that maximises display of the stigmatic lobes (Fig. 2B, E). In *L. aestiva*, the column is long, free from the corolla tube and held under tension in untriggered flowers, moving rapidly when released from the labellum and coming to an abrupt stop when it hits the sheath. This catapult-like mechanism results in pollen being sprayed outwards from the anthers (and presumably onto a pollinator). In this species, the stigmatic lobes mature once pollen has been shed, thereby promoting outcrossing or geitonogamous self-pollination. The stigmatic lobes in *L. pauciflora*, *L. preissii* and *L. pulcherrima* similarly appear to mature once pollen has been shed.

In *L. leptantha*, the column is adnate to the anterior side of the corolla tube, with only the short (0.7–1.1 mm long), distal portion free to move. Despite this, a catapult-like action is still achieved. The distal portion, which is slightly forward-arched and therefore held under a degree of tension by the labellum, moves rapidly to the opposite (posterior) side of the flower when the labellum is triggered, with its forward momentum apparently halted by its attachment to the anterior side of the corolla tube (the posterior rim of the column sheath may also act as a stopper). Like *L. aestiva*, pollen is sprayed outwards from the anthers, but may also spill onto the lower stigmatic lobe which, in contrast to *L. aestiva*, develops before the upper one and usually while the column is still enclosed by the labellum. The lower stigmatic lobe, which is strongly upturned, can often be seen protruding from the labellum and this may enable it to receive pollen from a visiting insect. The upper stigmatic lobe matures once the column has been exposed from the labellum and is prominently displayed to promote outcrossing. Staggered development of the stigmatic lobes is also evident in *L. chippendalei*, *L. dubia*, *L. octomaculata* and *L. stipitata*, of which *L. dubia* is akin to *L. leptantha* in having the column adnate to the corolla tube (a trait also shared with *L. pauciflora*, *L. pulcherrima* and possibly *L. sonderi*).

*Levenhookia pauciflora* has a distinct column morphology that generates a unique movement. In addition to adhering to the corolla tube, the column is attached to the anterior side of the column sheath, which further restricts its movement upon release from the labellum. Its distal end (Fig. 2H) is sharply angled towards the labellum and has a dilated tip subtended by a weak hinge. The hinge provides the tip with a degree of flexibility that may enable direct placement of pollen on an insect. Erickson (1958) noted that this species produced “a veritable shower” of pollen when triggered; however, I observed its column to move comparatively slowly into a nearly upright position without generating an obvious spray of pollen, although some pollen was transferred to the prominent tuft of hairs at the apex of the labellum, potentially functioning as a form of secondary pollen presentation.

Erickson (1958) suggested that the release of the column from the labellum in *Levenhookia* results in geitonogamous self-pollination via the catapulting of pollen between flowers on the same plant. This is at odds with my own observations, which indicate that the flowers are usually too widely spaced and pollen is not flung far enough for this to occur. Furthermore, in some species (*L. dubia*, *L. pauciflora*, *L. octomaculata* and *L. stipitata*), I observed little to no pollen spray; however, further observations are warranted, particularly given the suggestion that the column is held under increasing tension as the flower matures (Erickson 1958: 206). This suggests that variation is to be expected when studying flowers within a population or even on the same plant.

The interaction between the labellum and the column, which is difficult to investigate in the field given the size of the plants and the rapidity of the movements, remains incompletely studied. Observations are lacking for many species and pollination records are largely wanting. Erickson (1958: 203) noted woolly bee-flies actively probing flowers of *L. leptantha*, noting that pollen grains were “sprayed about the head and under the body”. I have only made two incidental observations: a small native bee on *L. pulcherrima* (at *J.A. Wege 1937*), moving rapidly between flowers while foraging for pollen and a large wasp repeatedly visiting *L. octomaculata* (at *J.A. Wege 2074*), although it was unclear whether it was transferring pollen between flowers.

#### Other notes.

Erickson (1958: 200) notes that *Levenhookia* flowers close at night by folding the four main corolla lobes together. I have observed this phenomenon in *L. aestiva*, *L. dubia*, *L. leptantha* and *L. octomaculata*; however, this trait is not universally exhibited, with the small flowers of *L. murfetii* and *L. pusilla* remaining open.

Mildbraed (1908) recognised three sections based on labellum and column sheath morphology (at which time only six species were recognised). A revised infrageneric classification will be considered once a robust molecular phylogenetic framework is available, although this is unlikely to be warranted given the size of the genus.

### A key to the species of *Levenhookia*

Note that column length can be estimated on pressed material by measuring from the tip of an exposed column to the base of the calyx lobes.

**Table:** 

1	Stem with glandular hairs and minute (< 0.1 mm) papillae (the latter readily visible towards the stem base); corolla lobes predominantly creamy white with a fine red mid-vein on the undersurface, posterior pair acuminate, acute or apiculate [W.A.]	**7. *L. pauciflora***
–	Stem glabrous or glandular-hairy, papillae absent; corolla lobes predominantly white and without a coloured midvein, or predominantly pink and rarely with a dark pink midvein, posterior pair obtuse, retuse, emarginate or bluntly pointed	**2**
2	Corolla tube longer than the longest calyx lobes	**3**
–	Corolla tube ± equal to or shorter than the longest calyx lobes	**7**
3	Labellum 0.6–2 mm long, sessile or with a short claw to ca. 0.2 mm long, apical appendage absent, to 0.5 mm long or with a tuft of white hairs; column adnate to corolla tube, with the top 0.5–2 mm free; flowering Aug–Nov	**4**
–	Labellum 3–6.5 mm long with a claw 1.2–2.5 mm long, apical appendage 0.7–2 mm long; column free to base; flowering late Oct-early Apr	**6**
4	Corolla tube 1–3 mm long, ± equal to or just longer than the calyx lobes; column 1.3–2.5 mm long; corolla lobes white or pale pink, 1.2–3 mm long; labellum without an apical appendage or tuft of hairs [W.A, S.A., N.S.W., Vic., Tas.]	**4. *L. dubia***
–	Corolla tube 3–9 mm long, exserted well beyond the calyx lobes; column 4.2–10 mm long; corolla lobes usually bright pink (rarely white), 1.8–5.5 mm long; labellum apical appendage or hair tuft present	**5**
5	Calyx lobes 0.8–2 mm long; labellum with rounded basal appendages and a small, papillate apical appendage; column sheath yellow, fused with the posterior corolla lobes to form a flat pad [W.A.]	**5. *L. leptantha***
–	Calyx lobes 1.8–3.5 mm long; labellum with linear-subulate basal appendages and a tuft of hairs at the apex; column sheath creamy white, with a narrowly triangular lobe to 1 mm high on the posterior side [W.A.]	**6. *L. pulcherrima***
6	Corolla tube 2–4.5 mm long; labellum 3–4.2 mm long with rounded basal appendages; column 4.5–7.2 mm long; column sheath white, lopsided, fused to posterior corolla lobes [W.A.]	**8. *L. preissii***
–	Corolla tube 5.5–8 mm long; labellum 4.5–6.5 mm long with oblong-subulate basal appendages; column 7.5–11 mm long; column sheath pink, entire, distinct from corolla lobes [W.A.]	**9. *L. aestiva***
7	Stem glabrous or with glandular hairs restricted to the distal portion	**8**
–	Stem glandular-hairy throughout length (rarely sparse toward the base)	**9**
8	Primary stem axis glabrous; outermost floral bracts glabrous; corolla lobes pink with a white base (rarely all white) [W.A., S.A., Vic.]	**1. *L. pusilla***
–	Primary stem axis glandular-hairy near the inflorescence; outermost floral bracts glandular-hairy abaxially towards the base; corolla lobes white or pink with pink-red markings near the base [W.A.]	**2. *L. murfetii***
9	Labellum 0.7–1.2 mm long, sessile or with a short claw to 0.2 mm long, lacking an apical appendage; column 1–2.5 mm long	**10**
–	Labellum 2–6 mm long with a claw 0.9–2 mm long, apical appendage present; column 2–4 mm long	**11**
10	Labellum hood yellow to whitish or sometimes with pinkish red markings, basal appendages absent or rudimentary; calyx lobes ± equal length, acute; corolla tube ± equal to or longer than the calyx lobes; hypanthium glandular-hairy [W.A., S.A., N.S.W., Vic., Tas.]	**4. *L. dubia***
–	Labellum hood dark red-purple (especially when dried), basal appendages linear-subulate; calyx lobes unequal in length, obtuse or subacute; corolla tube shorter than the longest calyx lobes; hypanthium with both glandular and eglandular hairs [S.A., Vic.]	**3. *L. sonderi***
11	Stem usually much-branched near base (rarely simple); leaves basal and cauline; labellum 4–6 mm long with a prominent, emarginate or incised, glabrous apical appendage [W.A., NT]	**12. *L. chippendalei***
–	Stem simple or branched along length; leaves cauline; labellum 2–3.7 mm long, with a small, blunt, papillate apical appendage	**12**
12	Floral bracts glabrous or with a few glandular hairs near the base; calyx lobes with very sparse glandular hairs mostly near the base; corolla lobes with 2 dark red-pink, ± rounded to elliptic markings toward the base (rarely faint or lacking?); column sheath up to half the length of the column [W.A.]	**11. *L. octomaculata***
–	Floral bracts glandular-hairy on the abaxial surface and margins; calyx lobes moderately to sparsely glandular-hairy throughout length; corolla lobes without markings or sometimes with 2 red-pink, ± oblong to linear markings toward the base; column sheath more than half the length of the column [W.A.]	**10. *L. stipitata***

### 1. Levenhookia pusilla

Taxon classificationPlantaeAsteralesStylidiaceae

R.Br., Prodr. Fl. Nov. Holland. 573. 1810

400D95E5-32B0-5AEE-BE5E-0F6C4E090B54

Figs 1C , 3B

Leeuwenhoekia pusilla , orth. var.: A.P. De Candolle, Prodr. 7: 338. 1839.
Leeuwenhookia pusilla , orth. var.: O.W. Sonder in J.G.C. Lehmann, Pl. Preiss. 1(3): 392. 1845.
Leewenhoekia pusilla , orth. var.: F. von Mueller, Syst. Census Austral. Pl.: 86. 1882.

#### Type.

**Australia. Western Australia**: near the observatory, Princess Royal Harbour, King George’s Sound, 21 Dec 1801, *R. Brown s.n.* [Bennett No. 2613] (lectotype, designated by Wege (2017: 231): BM 001041273; isolectotype: BM 000948765).

#### Description.

Annual herb 1–10 cm high. Glandular hairs ca. 0.1–0.2 mm long. Stem dark red, simple or branched to varying degrees with porrect lateral branches, mostly glabrous, a few glandular hairs sometimes present distally on the lateral branches. Leaves cauline, scattered, green adaxially, dark red or purplish red abaxially; lamina oblanceolate to narrowly oblanceolate, reniform, ovate or elliptic, 1.5–15 mm long including the petiole, 1–6.5 mm wide, obtuse or subacute, glabrous or scarcely papillate. Flowers in corymbs, usually crowded amongst the bracts, 1–500^+^ per plant; bracts narrowly oblanceolate to oblanceolate or linear, 1.8–9 mm long, usually glabrous, sometimes scarcely papillate on the margins, the upper-most bracts sometimes with a few glandular hairs abaxially towards the base; pedicels 0.3–2.5 mm long, sparsely glandular-hairy. Hypanthium globose, ellipsoid or ovoid, 0.7–1 mm long, 0.6–0.9 mm wide, with glandular hairs throughout and eglandular hairs 0.2–0.6 mm long distally. Calyx lobes subequal (with the anterior pair scarcely longer than the rest), 1–1.7 mm long, acute or subacute, glabrous or sparsely glandular-hairy towards the base, sometimes scarcely papillate apically. Corolla pink with a white base or occasionally all white, glabrous; lobes evenly arranged or sometimes ± paired vertically, distally recurved, obovate, ± equal in size or with the anterior pair scarcely longer than the posterior ones, 0.9–1.5 mm long, 0.6–0.9 mm wide, rounded or scarcely apiculate; tube white, 0.3–0.6 mm long, shorter than the calyx lobes. Labellum dorsal or sometimes ventral (rarely lateral), 0.7–0.8 mm long including a 0.1–0.2 mm long claw; hood dark red-pink or red-purple, usually sparsely glandular-hairy abaxially, minutely papillate adaxially along the margins; appendage at the cleft apex red-pink, ca. 0.1–0.2 mm long, papillate; basal appendages white, linear-subulate, ca. 0.3 mm long. Column sheath creamy white, glabrous, irregularly lobed, to 0.3 mm high, pendulous appendages absent. Column white, often tipped red-pink, free, 1–1.5 mm long, distally broadened and angled toward labellum, glabrous; stigmatic lobes to 0.4 mm long, developing while the column is hooded, erect to incurved. Capsule ovoid, 1.3–3 mm long excluding calyx lobes. Seeds 0.4–0.7 mm long, 0.3–0.4 mm wide.

#### Diagnostic features.

*Levenhookia pusilla* has a dark red and glabrous primary stem axis, glabrous or scarcely papillate leaves and outermost bracts that are usually green adaxially and dark red or purplish red abaxially, and tiny flowers with a pink and white (occasionally all white) corolla with lobes 1–1.5 mm × < 1 mm.

#### Phenology.

Flowering from mid-September to early December; fruiting from October to early January.

#### Distribution.

**Figure 3.**
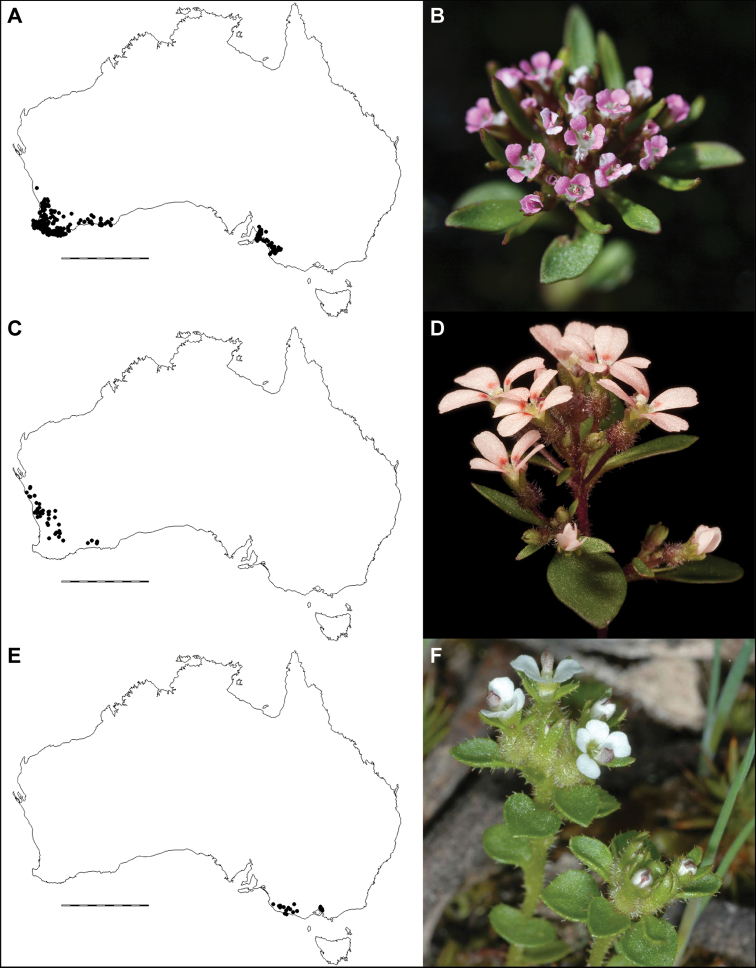
Comparative distributions and floral morphologies **A, B***Levenhookia pusilla*, with a disjunct distribution in Western Australia and South Australia and a dense inflorescence of pint-sized flowers (unvouchered, Mt Clarence, Albany, Western Australia) **C, D***L. murfetii*, a Western Australian endemic with discrete markings at the base of the corolla lobes (*J.A. Wege 1829*) **E, F***L. sonderi*, a rarity from South Australia and Victoria with red-purple markings on the labellum that are especially prominent in pressed material (unvouchered, from near St Andrews, Victoria). Photos by J.A. Wege (**B**), K.R. Thiele (**D**) and C. Lindorff (**F**). Scale bar on maps 1000 km.

*Levenhookia pusilla* has a disjunct distribution in southern Australia (Fig. 3A). In south-western Australia, it is common in the Jarrah Forest, Warren and Swan Coastal Plain bioregions and scattered across the southern Avon Wheatbelt, Mallee and Esperance bioregions, with one outlying record from the Geraldton Sandplains. In south-eastern Australia, it is restricted to the Flinders Lofty Block, Eyre York Block, Murray Darling Depression, Naracoote Coastal Plain and Kanmantoo bioregions in south-eastern South Australia, extending into Victoria at Little Desert National Park near the South Australian border.

#### Habitat.

This species grows in sand or loamy sand on ridges, hill-slopes, plains or dune swales, often with lateritic gravel or in association with granite outcropping; it is more rarely recorded growing in clay or clay loam in depressions or seasonally-wet flats. Associated vegetation is varied and includes tall *Eucalyptus* woodland or forest, low open woodland with *Eucalyptus*, *Allocasuarina* or *Melaleuca*, shrubland or scrub with *Banksia*, *Melaleuca* or emergent mallees, and low heath. It commonly co-occurs with *L. stipitata* in Western Australia and is often abundant on firebreaks and along track edges.

#### Conservation status.

*Levenhookia pusilla* is widespread and locally abundant across most of its range (IUCN (2012): Least Concern), but it is listed as Vulnerable in Victoria (Department of Environment and Primary Industries, Victoria 2014), where it is restricted to Little Desert National Park.

#### Etymology.

From the Latin *pusillus* (very small); an aptly named plant given its tiny flowers and diminutive habit.

#### Vernacular name.

Midget Stylewort (Erickson 1958).

#### Notes.

*Levenhookia pusilla* is morphologically allied to *L. murfetii* (refer to the comparative notes under that species) and the two species are known to grow near one another in Western Australia’s Avon Wheatbelt. It often has a dorsally positioned labellum, a feature shared with *L. murfetii* and achieved through rotation of the pedicel; however, a ventral or lateral labellum has also been observed in instances where the crowded flowers and bracts prevent rotation or full rotation of the pedicels.

A specimen of *L. pusilla* from the Geraldton Sandplains bioregion near Warradarge (*M. Hislop 1492*, PERTH) is a northern outlier: further collections and observations from this region would be of interest, particularly given the widespread occurrence of *L. murfetii* in this area.

A subset of individuals on the following three specimens of *L. pusilla*, which are from three separate locations in South Australia, have been infected by a smut: *R. Bates 51370* (AD), *A.G. Spooner 1619* (AD) and *D.J.E Whibley 10106* (AD). A smut has also been detected on collections of *L. sonderi* (refer to the notes under that species). No smuts have been formally identified on Stylidiaceae to date (Shivas et al. 2014).

#### Illustrations.

F. Bauer, Ill. Fl. Novae Holl., t. 15. 1816 [corolla lobes inaccurately depicted]; R. Erickson, Triggerplants 201, Pl. 57, No. 1. 1958; B.J. Grieve & W.E. Blackall, How to know W. Austral. wildfl. 4: 765, No. 3. 1982; H.R. Toelken in J.P. Jessop & H.R. Toelken, Fl. South Austral. 1419, fig. 639b. 1986; E.J. Raulings in N.G. Walsh & T.J. Entwisle, Fl. Victoria 4: 583, fig. 111a. 1999 [the only cited illustration to correctly depict the presence of both eglandular and glandular hairs on the hypanthium]; J. Wheeler, N. Marchant & M. Lewington, Fl. South West 2: 902. 2002.

#### Selected specimens examined.

**Australia. Western Australia**: Mt Merivale, 20 km E of Esperance, 29 Nov 1997, *B. Archer 911* (MEL); Brixton Street Wetlands, Kenwick, 10 Oct 2007, *K.L. Brown & G. Paczkowska KLB 673* (PERTH); Tutanning Reserve, SE of Pingelly, 7 Oct 1973, *A.S. George 11712* (PERTH); Julimar forest, corner West Boundary Rd and 7 Mile Rd, 1 Oct 2001, *M. Hislop 2319* (PERTH); Bramley National Park, NW Margaret River, off Burnside Rd, 19 Nov 2008, *G.J. Keighery 17437* (PERTH); Mira Flores Ave, off Millinup Rd, near Porongurup Range, ca. 3 km W of Porongurup, 31 Oct 1995, *T.R. Lally 821* (PERTH); Torbay Hill, West Cape Howe National Park, 16 Nov 1995, *T.R. Lally & B.J. Lepschi 913* (PERTH); 50 m N on track off Quartz Rd, ca. 400 m from Coronation Rd, W of Manjimup, 9 Nov 2002, *J.A. Wege 795* (PERTH); Near inlet campsite, Waychinicup National Park, 28 Oct 2003, *J.A. Wege 1051 & C. Wilkins* (PERTH); Margaret River Rd, E of Great N Rd, E of Margaret River, 8 Nov 2009, *J.A. Wege 1749 & W.S. Armbruster* (K, MEL, PERTH); 1.1 km W of Stan Rd on Blue Lake Rd, SW of Mt Barker, 24 Oct 2018, *J.A. Wege 2072 & C. Wilkins* (MEL, PERTH); **South Australia**: Noolook Forest Reserve, 15 Oct 1984, *N.N. Donner 10273* (AD, MEL); Myponga Conservation Park, 14 Oct 1986, *D.E. Murfet 228* (AD); Newland Head Conservation Park, 29 Sep 2009, *D.E. Murfet 6603* (AD); Cox Scrub Conservation Park, 27 Sep 2008, *K.A. Shepherd & J.A. Wege KAS 1139* (PERTH); **Victoria**: Little Desert, 11 Oct 1989, *J.G. Eichler s.n.* (MEL).

### 2. Levenhookia murfetii

Taxon classificationPlantaeAsteralesStylidiaceae

Lowrie & Conran, J. International Triggerplant Society 1(2): 14–16, figs 16–19, 48I. 2011

AC30583C-D71A-5B2F-A671-37DD78139E05

Figs 2I , 3D

Levenhookia pusilla auct. non R.Br.: R. Erickson, Triggerplants 207–209 (1958), *p.p.*

#### Type.

**Australia. Western Australia**: Brand Highway near Marchagee Road turn-off, Coomallo, 11 Sep 2007, *A. Lowrie 3553 & J.G. Conran* (holotype: PERTH 08298262; isotype: MEL 2385577).

#### Description.

Annual herb 1–9 cm high. Glandular hairs 0.1–0.4 mm long. Stem dark red, simple or branched to varying degrees with porrect lateral branches, glabrous basally, glandular-hairy distally. Leaves cauline, scattered, green adaxially, reddish or green abaxially; lamina oblanceolate to narrowly oblanceolate, reniform or ovate, 2.5–13 mm long including the petiole, 1–5 mm wide, obtuse or subacute, glabrous or scarcely papillate, the uppermost leaves sparsely glandular-hairy abaxially towards the base. Flowers in corymbs, 1–ca. 40 per plant; bracts narrowly lanceolate to lanceolate or linear, 2.5–10 mm long, sparsely glandular-hairy abaxially towards the base, sometimes scarcely papillate on the margins; pedicels 1–4 mm long, sparsely glandular-hairy. Hypanthium depressed globose, globose or ovoid, 0.7–1.2 mm long, 0.7–1.4 mm wide, with glandular hairs throughout and sparse eglandular hairs 0.15–0.5 mm long distally. Calyx lobes subequal (with the anterior pair scarcely longer than the rest), 0.9–1.7 mm long, acute to subacute, sparsely glandular-hairy in lower 1/2–2/3, usually scarcely papillate apically. Corolla pale to medium pink or white, with red-pink throat markings and a white or yellowish green throat, glabrous; lobes ± paired vertically to somewhat evenly arranged, spreading to scarcely recurved, rounded to scarcely emarginate or apiculate; anterior (upper) lobes elliptic to narrowly obovate, slightly inwardly curved, ± equal to or a little shorter than the posterior lobes, 1–1.8 mm long, 0.6–1.2 mm wide; posterior lobes obovate, 1.2–2 mm long, 0.8–1.3 mm wide; tube white or yellowish, 0.5–0.7 mm long, shorter than the calyx lobes. Labellum dorsal, 0.7–0.8 mm long including a 0.1–0.2 mm long claw; hood dark red-pink, usually sparsely glandular-hairy abaxially, minutely papillate adaxially along the margins; appendage at the cleft apex bright pink, ca. 0.1–0.3 mm long, papillate; basal appendages white to yellowish, linear-subulate, 0.2–0.3 mm long. Column sheath creamy white to yellowish, glabrous, lopsided, with a narrowly triangular posterior lobe to 0.3 mm high and slightly smaller lateral lobes, pendulous appendages absent. Column whitish, free, 1–1.4 mm long, distally broadened and angled towards labellum, glabrous; stigmatic lobes to 0.5 mm long, developing while the column is hooded, erect to incurved. Capsule ovoid, 1.3–2.2 mm long excluding calyx lobes. Seeds 0.4–0.5 mm long, 0.3–0.4 mm wide.

#### Diagnostic features.

*Levenhookia murfetii* has a stem that is glabrous basally and glandular-hairy distally, bracts with glandular hairs restricted to the undersurface, and a pink or white corolla with small lobes (1–2 mm long) bearing a small red-pink marking towards the base.

#### Phenology.

Flowering from late August to early October; fruits have been collected in October.

#### Distribution.

*Levenhookia murfetii* is endemic to south-western Australia (Fig. 3C) where it occurs in the Geraldton Sandplains, Avon Wheatbelt and Mallee bioregions, from Eurardy Station to east of Grass Patch.

#### Habitat.

This species usually grows in sand or sandy loam (rarely in clay loam), often with lateritic gravel (rarely with decomposed granite). It is commonly recorded in low heath, mallee shrubland, tall *Allocasuarina* or *Melaleuca* shrubland, and *Eucalyptus wandoo* woodland. It is often abundant on firebreaks or near the base of open shrubs.

#### Conservation status.

This widespread species is locally abundant at a range of sites (IUCN 2012: Least Concern).

#### Etymology.

Honours Denzel E. Murfet, who is affiliated with the State Herbarium of South Australia and has made more than 650 Stylidiaceae collections from across Australia (AVH 2019).

#### Vernacular name.

Kwongan Stylewort.

#### Notes.

Lowrie and Conran (2011) described *L. murfetii* from a limited number of specimens collected from north and north-east of Perth. Examination of collections at PERTH indicate a much broader geographic range and reveal that some of the features they used to separate it from *L. pusilla* are not taxonomically informative, most notably whether the stem is simple or branched, labellum morphology and inflorescence structure. The simplest way to distinguish pressed material of these two species is by examining the distal portion of the stem and the abaxial surface of the outermost floral bracts, which are always glandular-hairy in *L. murfetii* and glabrous in *L. pusilla*. *Levenhookia murfetii* mostly flowers earlier in the season (from late August to early October cf. late September to early December) and its flowers tend to have more openly-spread corolla lobes (compare Fig. 3B, D) with discrete red-pink markings near the base (Fig. 2I) (markings absent in *L. pusilla*). While individual plants of *L. murfetii* often produce fewer flowers than in *L. pusilla*, flower number cannot be used to reliably separate these two species. They are largely geographically separated, although an apparent mixed collection from the Bolgart area (*R. Erickson s.n.*, PERTH 02769182) suggests that they may occur in sympatry. They are known to grow in proximity to one another in the Tarin Rock and Toodyay areas and probably also near Warradarge (refer to the notes under *L. pusilla*).

#### Illustrations.

R. Erickson, Triggerplants 208, Pl. 58, Nos. 1–7. 1958, as *L. pusilla*.

#### Selected specimens examined.

**Australia. Western Australia**: junction of Yerina Springs Rd and Ogilvie Rd, 15 km NNE of Gregory, 11 Sep 2004, *R.K. Brummitt 21236*, *A.S. George & E.G.H Oliver* (PERTH); due E of N end of Corry Rd, W of Corrigin, 24 Sep 2007, *M. Hislop & M. Griffiths WW 209 – 27* (PERTH); 21 miles [33.8 km] N of Geraldton, Moresby Range, 25 Aug 1974, *D. & N. McFarland 1137* (PERTH); Mt Lesueur National Park, 9 Sep 2008, *D.E. Murfet & A. Lowrie DEM 6345* (AD, PERTH); SE corner of Reserve 24952, 23 Sep 1998, *E.M. Sandiford 326* (PERTH); 750 m E along Hills Rd from the Lake Grace – Dumbleyung Rd, Tarin Rock Nature Reserve, 21 Sep 1997, *J.A. Wege 360*, *R. Butcher & C. Wilkins* (PERTH); 6.9 km S of Coorow – Greenhead Rd on Midlands Rd, Marchagee Nature Reserve, 16 Sep 2011, *J.A. Wege 1829 & K.R. Thiele* (PERTH); Elphin Nature Reserve off Waddington – Wongan Hills Rd, 11 Sep 2018, *J.A. Wege 2060* (CANB, MEL, PERTH); ca. 4.4 km E of First North Rd on Eneabba – Three Springs Rd, Wotto Nature Reserve, 13 Sep 2018, *J.A. Wege 2066* (PERTH); 2.5 km W from Brand Hwy on Coorow – Greenhead Rd, 14 Sep 2018, *J.A. Wege 2067* (CANB, MEL, PERTH).

### 3. Levenhookia sonderi

Taxon classificationPlantaeAsteralesStylidiaceae

(F.Muell.) F.Muell., Fragm. 1(1): 18. 1858, as Leeuvenhookia

E70F2DA6-AE52-52F8-8CFE-924F057A163C

Fig. 3F

Coleostylis sonderi F.Muell., Second systematic index of the plants of Victoria. Victoria, Parliamentary Paper no. A 18: 13. 1854, *nom. nud.*
Coleostylis sonderi F.Muell., Definitions of rare or hitherto undescribed Australian plants 13. 1855 [see O. Seberg, Taxon 35: 267 (1986) for publication date].
Coleostylis sonderi F.Muell., Trans. Philos. Soc. Victoria 1: 46. 1855, isonym.
Leewenhoekia sonderi , orth. var.: F. von Mueller, Syst. Census Austral. Pl.: 86. 1882.
Levenhookia dubia var. sonderi (F.Muell.) Mildbr. in H.G.A. Engler, Pflanzenr. 35: 27. 1908.

#### Type.

**Australia. Victoria**: Violet Creek, *C. Wilhelmi s.n.* (lectotype, here designated: MEL 1617988 [the individual mounted on the sheet together with those in the large, cream, rectangular packet annotated “Levenhookia Sonderi, Mueller’s”]; isolectotypes: K 000060054, MEL 1617989, MEL 2256398, TDC [3 individuals on right]).

#### Description.

Annual herb 1–10 cm high. Glandular hairs 0.1–0.7 mm long. Stem pale, green, straw brown or more rarely reddish brown, simple or occasionally with porrect lateral branches, sparsely glandular-hairy. Leaves cauline, scattered, pale green; lamina ovate or orbicular, 1–8 mm long including the petiole, 1–4.5 mm wide, subacute or obtuse, sparsely glandular-hairy abaxially and on the margins. Flowers usually in corymbs or umbels, sometimes in short racemes, 1–ca. 23 per plant; bracts with an ovate or lanceolate lamina, 1–7 mm long including the petiole, glandular-hairy like the leaves; pedicels 0.5–10 mm long, sparsely glandular-hairy. Hypanthium globose, ovoid or ellipsoid, 0.7–2 mm long, 0.7–2 mm wide, with glandular hairs throughout and sparse eglandular hairs 0.4–0.8 mm long distally. Calyx lobes unequal (with the anterior pair longer than the rest), 0.7–1.7 mm long, obtuse or subacute, sparsely glandular-hairy. Corolla white with a yellowish green throat; lobes ± evenly arranged or with the upper (posterior) ones ± paired vertically, rounded or scarcely retuse, sparsely glandular-hairy abaxially; anterior lobes obovate, slightly longer and broader than the posterior pair, ca. 1.2–1.3 mm long, 0.8–0.9 mm wide; posterior lobes elliptic or obovate, ca. 0.7–1 mm long, 0.7–0.8 mm wide; tube yellowish green, 0.8–1.3 mm long, shorter than the longest calyx lobes, sparsely glandular-hairy distally. Labellum ventral, 0.8–1.2 mm long including a ca. 0.2 mm claw; hood dark red-purple, with sparse glandular hairs abaxially, lacking an appendage at the cleft apex; basal appendages white, linear-subulate, 0.3–0.4 mm long. Column sheath yellowish green, glabrous, to 0.3 mm high and irregularly lobed on anterior side, scarcely visible on posterior side, pendulous appendages absent. Column white, seemingly adnate to the anterior side of the corolla tube, 1.8–2.5 mm long with the top ca. 1 mm free, distally angled toward the labellum, glabrous; stigmatic lobes to ca. 0.5 mm long, apparently developing while the column is hooded, erect to scarcely incurved. Capsule globose, ovoid or ellipsoid, 2–3.5 mm long excluding calyx lobes. Seeds 0.6–1 mm long, 0.5–0.6 mm wide.

#### Diagnostic features.

*Levenhookia sonderi* has a pale, sparsely glandular-hairy stem, leaves with an ovate or orbicular lamina, glandular and eglandular hairs on the hypanthium, unequal calyx lobes with obtuse to subacute apices, and a red-purple labellum (appearing very dark on pressed material) with white, linear-subulate basal appendages. Its seeds are the largest in the genus.

#### Phenology.

Flowering September–December; fruiting October–December.

#### Distribution.

*Levenhookia sonderi* is endemic to South Australia and Victoria (Fig. 3E), where it is has been recorded from the Naracoote Coastal Plain, Southern Volcanic Plain, Victorian Midlands and South Eastern Highlands bioregions, extending from Reedy Creek (South Australia) in the west to the Dandenong Ranges (Victoria) in the east.

#### Habitat.

This species grows in damp sandy loam or clayey sand on hill-slopes or more commonly in lowland areas, including near the margins of swamps. It favours open patches or lightly-disturbed areas in open woodland with *Eucalyptus camaldulensis*, *E. macrorhynca*, *E. polyanthemos* or *E. goniocalyx*, or near stands of *Kunzea phylicoides*, and grows in association with other diminutive herbs including *L. dubia*, *Stylidium beaugleholei*, *S. despectum* and *S. perpusillum*.

#### Conservation status.

*Levenhookia sonderi* is listed as Rare in Victoria (Department of Environment and Primary Industries, Victoria 2014) and Vulnerable (Schedule 8) in South Australia (Government of South Australia 2018) and may warrant listing at the national level under the *Environment Protection and Biodiversity Conservation Act 1999*. Populations of this species are isolated and appear highly localised, with the reproductive capacity of some individuals from locations in South Australia and Victoria impacted by a species of smut (see notes below).

#### Etymology.

Honours German apothecary and botanist Otto Wilhelm Sonder (1812–1881), who described a suite of new taxa as part of his account of Stylidiaceae in Lehmann’s *Plantae Preissianae* (Sonder 1845).

#### Vernacular name.

Slender Stylewort (Erickson 1958).

#### Typification.

MEL 1617988, which is annotated by Mueller and was viewed by Bentham (1868) for *Flora Australiensis*, comprises a mounted individual and an envelope housing five separate packets that represent more than one gathering. The mounted individual and the numerous additional plants in the large, cream, rectangular packet annotated “Levenhookia Sonderi, Mueller’s” have been selected as an appropriate lectotype. This material is comparable to that of K 000060054, MEL 1617989 and MEL 2256398—they are at the same flowering stage and bear white mycelia, presumably from being poorly dried following their collection—and showcases the distinctive, dark labellum, which Mueller notes on the label (“labellum atropurpureum!”). Material at TCD is also interpreted as being part of this gathering.

The remaining material on MEL 1617988 is not treated herein as type material. There is a blue, rectangular packet annotated “Coleostylis sonderi Portland” that contains a single flowering plant, which Mueller may have separated from MEL 2256407 to send to Bentham. There is a smaller blue packet annotated “Leeuwenhoekiasonderi” that contains flower fragments, but it is unclear to which gathering they belong. Finally, there are two packets containing seeds and withered corolla fragments that must be from a separate collection to that of the type gathering, which is not in fruit. It is unknown whether these seeds are from the type population or from a different locality or, indeed, two separate localities. It is of note that there are no fruiting specimens at MEL that predate the publication of this species and Mueller did not describe seed in the protologue. Seed is also inexplicably present in the packet attached to Portland’s flowering collection (MEL 2256407).

#### Notes.

*Levenhookia sonderi* is most likely to be confused with *L. dubia*, a species with which it can co-occur (there have been mixed collections made of the two (e.g. *D.J. Van Bockel 195A* and *195B*, MEL; *H.B. Williamson s.n.*, MEL 2256403). Their superficial similarity led Mildbraed (1908) to treat *L. sonderi* as a variety of *L. dubia*, but it was reinstated as a distinct species by Erickson (1958) on account of its dark red-purple labellum hood (cf. cream to yellow or pale pinkish red), green stems (cf. reddish) and shorter corolla tube (0.9–1.3 mm long and shorter than the longest calyx lobes cf. 1.5–3 mm long and ± equal to or longer than the calyx lobes). While stem colour cannot be used to reliably separate the two species, labellum colour and corolla tube length are informative and can be observed on pressed material. *Levenhookia sonderi* can be further separated from *L. dubia* by its unequal and obtuse or subacute calyx lobes (cf. equal and acute), hypanthium indumentum of both glandular and eglandular hairs (cf. glandular-hairy), linear-subulate basal appendanges on the labellum (cf. appendages lacking or rudimentary and rounded) and larger seed (0.6–1 mm long cf. 0.25–0.4 mm). It also tends to have more rounded leaf apices than *L. dubia*.

The column of *L. sonderi* appears to be adnate to the corolla tube like that of *L. dubia*, although this requires verification given the limited spirit material available for study. In one dissected flower, both stigmatic lobes were developed under the labellum hood with copious pollen observed. Like *L. pusilla* and *L. murfetii*, this suggests autogamy; *L. sonderi* is certainly akin to these two species, with all three sharing an indumentum of both eglandular and glandular hairs on the hypanthium.

Some individuals on the following collections of *L. sonderi* from South Australia and Victoria are infected by a smut that proliferates in the ovary, preventing the production of flowers and seed: *R. Bates 32191* (AD); *D. Hunt 2229* (AD); *D. Hunt 2241* (AD, MEL); *H.B. Williamson s.n.* (MEL 2256399, MEL 275679).

#### Illustrations.

E.J. Raulings in N.G. Walsh & T.J. Entwisle, Fl. Victoria 4: 583, fig. 111b. 1999.

#### Selected specimens examined.

**Australia. South Australia**: [localities obfuscated for conservation reasons] N of Mt Gambier, 8 Nov 1964, *D. Hunt 2229* (AD); N of Mt Gambier, 5 Dec 1964, *D. Hunt 2241* (AD, MEL); Reedy Creek, 1 Oct 1998, *D.E. Murfet 3317* (AD); Reedy Creek, 27 Oct 2011, *D.E. Murfet 7404* (AD); [E of Nangwarry], 3 Nov 2013, *D.E. Murfet 7624* (AD); **Victoria**: S of Crawford River, 20 Nov 1964, *A.C. Beauglehole ALB 43334* (MEL); Dandenong Range, 20 Oct 1977, *M.G. Corrick 5976* (MEL); Hawkesdale, Nov 1899, *H.B. Williamson s.n.* (MEL 2256403A); Warrandyte, 23 Oct 1983, *J.Z. Yugovic s.n.* (MEL).

### 4. Levenhookia dubia

Taxon classificationPlantaeAsteralesStylidiaceae

Sond., in J.G.C. Lehmann, Pl. Preiss. 1(3): 392. 1845, as Leeuwenhookia

BC1E7DC4-EE71-5C2E-80EE-5E176B3CABD1

Figs 2J, K , 4B

Levenhookia creberrima F.Muell., Fragm. 3(21): 121. 1862, as Leeuwenhoekia. Type. Australia. South Australia: St Vinc[ent] Gulf, 1851, *F. Mueller s.n.* (syntype: MEL 2257614); Gipps Land, [no date] *F. Mueller s.n.* (syntype: K 000060105); Victoria, [no date] *F. Mueller s.n.* (possible syntype: GH 00033478 image!, MEL 2254071).
Leewenhoekia dubia , orth. var.: F. von Mueller, Syst. Census Austral. Pl.: 86. 1882.

#### Type.

**Australia. Western Australia**: In sublimosis humidis prope Bassandeen [Bassendean] ad fluvium cygnorum, [1838–1842] *L. Preiss 2252* (lectotype, here designated: MEL 2257601; isolectotypes: FI 006941, G 00358739 image!, G 00358740 image!, LD 1730835 image!, MEL 2412198, MEL 2257599, P 00712438 image!, S 06-3637, W); Swan River [1841], *J. Drummond 516* (syntypes: BM 000948743, G 00358738 image!, K 000060096, K 000060108, OXF, MEL 2257600, MEL 2257596, W [2 sheets])

#### Description.

Annual herb 1.2–9 cm high. Glandular hairs 0.1–0.5 mm long. Stem dark red or pale reddish to greenish brown, simple or with porrect lateral branches, glandular-hairy. Leaves cauline, scattered, green or reddish; lamina ovate, oblanceolate, lanceolate or elliptic, sometimes narrowly so, 1–10 mm long including the petiole, 0.5–3 mm wide, subacute to acute, glandular-hairy abaxially and on the margins, sometimes also adaxially near the base. Flowers in short racemes or corymbs, 1–40(–ca. 70) per plant; bracts oblanceolate, lanceolate or elliptic (sometimes narrowly so), 1.2–9 mm long, glandular-hairy like the leaves; pedicels 0.5–5(–10) mm long, glandular-hairy. Hypanthium depressed globose to globose, ellipsoid or obovoid, 0.7–2.2 mm long, 0.7–1.8 mm wide, glandular-hairy. Calyx lobes ± equal, 0.7–2.2 mm long, acute, glandular-hairy. Corolla white or more rarely pale pink, with a yellow throat, rarely with faint red-pink markings at the base the lobes; lobes ± equal and evenly arranged or with the anterior (lower) lobes scarcely longer and broader than the posterior pair, spreading to scarcely recurved, obovate or elliptic, 1.2–3 mm long, 0.8–2 mm wide, retuse or rounded, glabrous or with a few glandular hairs abaxially towards base; tube 1–3 mm long, ± equal to or up to 1.2 mm longer than the calyx lobes, white, sparsely glandular-hairy distally. Labellum ventral, 0.6–1 mm long, ± sessile; hood yellow to whitish, sometimes with pinkish red markings, sparsely glandular-hairy abaxially, without an appendage at the cleft apex; basal appendages absent or rudimentary and obtuse. Column sheath yellowish, glabrous, reduced to a scarcely lobed rim to ca. 0.2 mm high on the anterior side, connate with the posterior corolla lobes forming a smooth, thickened pad, pendulous appendages absent. Column creamy yellow, adnate to the anterior side of the corolla tube, 1.3–2.5 mm long with the top 0.5–0.8 mm free and gently forward-arched when enclosed by the labellum, glabrous; stigmatic lobes to ca. 0.3 mm long, the lower-most sharply upturned and developing while the column is hooded, the upper lobe incurved and developing later. Capsule globose, ovoid or obovoid, 1.2–2.2 mm long excluding calyx lobes. Seeds 0.25–0.4 mm long, 0.2–0.3 mm wide.

#### Diagnostic features.

*Levenhookia dubia* has a glandular-hairy stem, acute calyx lobes, a corolla tube roughly equal to or a little longer than the calyx lobes, and a ±sessile labellum that lacks an apical appendage.

#### Phenology.

Flowering from August to November, with fruits recorded from mid-September to November.

#### Distribution.

**Figure 4.**
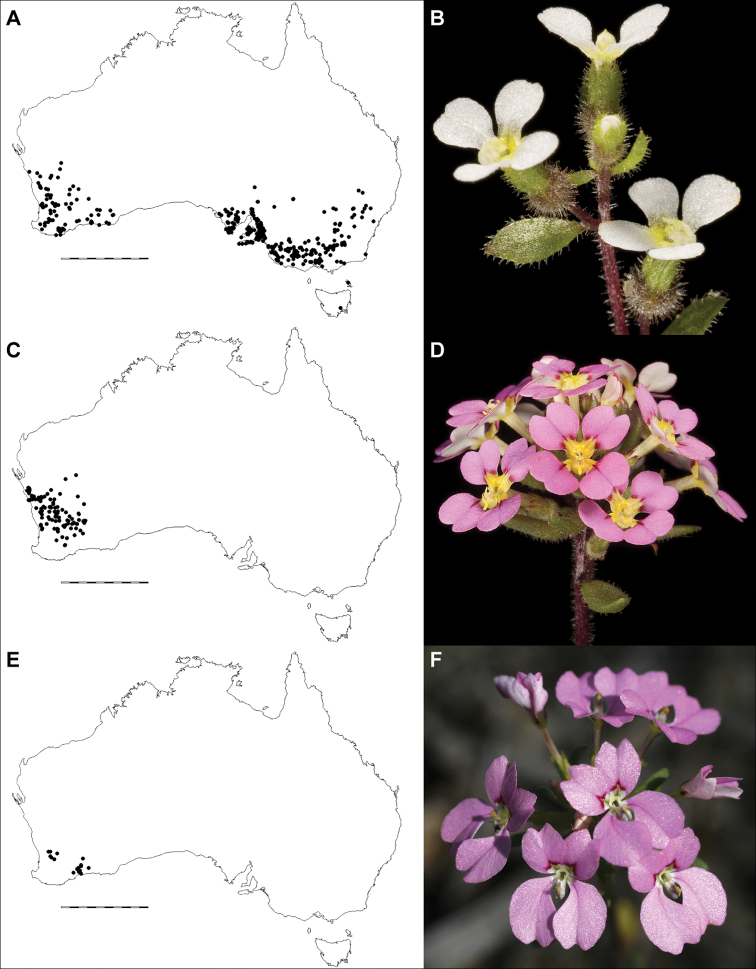
Comparative distributions and floral morphologies **A, B***Levenhookia dubia*, with a widespread distribution across southern Australia and flowers with acute calyx lobes and a simple, sessile labellum (*K.R. Thiele 3360*) **C, D***L. leptantha*, a widespread Western Australian endemic with an exserted corolla tube and fleshy bracts (*J.A. Wege 1828*) **E, F***L. pulcherrima*, a poorly-known Western Australian endemic with an exserted corolla tube and distinctive corolla lobe shape and markings (*J.A. Wege 1937*). Photos by K.R. Thiele (**B, D**) and J.A. Wege (**F**). Scale bar on maps 1000 km.

*Levenhookia dubia* is widespread across southern mainland Australia, with records spanning near coastal areas to the semi-arid interior (Fig. 4A). There are several historical collections from mainland Tasmania and Flinders Island from the 1800s (see additional information under ‘Conservation status’).

#### Habitat.

*Levenhookia dubia* grows in sand, sandy loam or clay loam, often in depressions or shallow drainage areas, near creek-lines or in damp soils associated with rocky outcropping including granite monoliths. It commonly grows with other ephemeral herbs, often in open *Eucalyptus* forest or mallee woodland, or shrubland with *Acacia*, *Allocasuarina*, *Callitris* or *Melaleuca*.

#### Conservation status.

This widespread species is not currently considered to be at risk (IUCN 2012: Least Concern); however, it is currently listed as extinct in Tasmania under the *Tasmanian Threatened Species Protection Act 1995* (Tasmanian Government 2019). Although historically recorded from Brighton, Flinders Island and Mount Field (Spicer 1878; Rodway 1903), the sole collection from this State at the Tasmanian Herbarium is one by William Archer that lacks locality information; accordingly, the veracity of these early works has been questioned (e.g. Gray 2011). I have recently viewed material collected in Tasmania at the National Herbarium of Victoria, specifically, collections by William Spicer (from Pontville near Brighton: MEL 2257574, 2257575 and 2257577) and Joseph Milligan (from the Strzelecki Peaks on Flinders Island: MEL 2257576) that support a distribution that includes Tasmania and provide information for future survey efforts. Appropriately timed searches in the Strzelecki National Park that focus on herb fields, moss beds or open runoff areas associated with granite outcropping, may lead to its rediscovery. In the absence of a corresponding voucher, the Mount Field locality of Rodway (1903) cannot be confirmed and is interpreted as a likely error.

#### Etymology.

From the Latin *dubius* (doubtful), alluding to Sonder’s (1845) uncertainty with regards to its generic classification.

#### Vernacular name.

Hairy Stylewort (Erickson 1958).

#### Typification.

Sonder examined and annotated specimens of *L. dubia* in his personal herbarium (now at MEL), as well as material at BM and LD, all of which conform to the protologue. The designated lectotype (MEL 2257601) is the best quality material.

#### Notes.

In south-eastern Australia, *L. dubia* may be confused with *L. sonderi* (refer to the comparative notes under that species).

#### Illustrations.

F. von Mueller, Pl. Indig. Victoria Pl. 48. 1865, as *Leeuwenhoekia creberrima*; B.J. Grieve & W.E. Blackall, How to know W. Austral. wildfl. 4: 765, no. 1. 1982; H.R. Toelken in J.P. Jessop & H.R. Toelken, Fl. S. Austral. ed. 4: 1419, fig. 639A. 1986; G.R.M. Dashorst & J.P. Jessop, Plants of the Adelaide plains and hills 138, fig. 9. 1990; E.J. Raulings in N.G. Walsh & T.J. Entwisle, Fl. Victoria 4: 583, fig. 111c. 1999; J. Wheeler, N. Marchant & M. Lewington, Fl. South West 2: 902. 2002; L.C. Stanberg in G. Harden, Fl. New South Wales 3: 12. 1992.

#### Selected specimens examined.

**Australia. Western Australia**: Garden Rock 16 km along Cue and Sandstone road, 17 Aug 2003, *G. Byrne 371* (PERTH); Pigeon Rocks, Sep 1973, *S. James 73.9/13* (PERTH); Mt Willgonarinya, ca. 72 km S of Balladonia Motel, Eyre Highway, 15 Sep 1980, *K.R. Newbey 7403* (PERTH); Location 1105, ca. 22 km N of Coast at Stokes Inlet, 27 Sep 1968, *A.E. Orchard 1220* (AD, PERTH); The Humps, near Hyden, 13 Sep 1983, *R. Ornduff 9309-15* (PERTH); Woody Island, 5 Oct 2003, *E. Rippey 582* (PERTH); 100 m S of Gura-Beekeepers Road junction, 3 Oct 1988, *D. Rose 710* (PERTH); Dingo Rock, 11 km W of Manmanning, 7 Sep 2002, *B.H. Smith 2010* (MEL); Quairading Nature Reserve, 27 Sep 2007, *K.R. Thiele 3360* (PERTH); granite outcrop near Phillips River crossing on Aerodrome Road, NW of Ravensthorpe, 28 Sep 1997, *J.A. Wege 376*, *R. Butcher & C. Wilkins* (PERTH); granite apron, E side of Mt Hampton, 20 Sep 2010, *J.A. Wege 1773 & C. Wilkins* (K, MEL, PERTH); 8.9 km S of Talbot West Road on Yarra Road, WSW of York, 25 Sep 2011, *J.A. Wege 1836 & B.P. Miller* (PERTH); S of Thomas River mouth, Cape Arid National Park, 10 Oct 2011, *J.A. Wege 1860 & C. Wilkins* (PERTH); N of Koorda – Cadoux Rd, just E of Rabbit Proof Fence Rd, Noorajin Soak Nature Reserve, 11 Sep 2018, *J.A. Wege 2062* (PERTH); **South Australia**: ca. 15 km W of Murray Bridge, 2 Oct 1974, *J. Carrick 3826* (AD); ca. 4 km NE of Weetulta, 29 Sep 1968, *B. Copley 2292* (AD); Waterfall Gully, ca. 9.5 km SE of Adelaide, 14 Oct 1957, *Hj. Eichler 14309* (AD); Carappee Hill Conservation Park, 24 Sep 2003, *P.J. Lang & P.D. Canty BS128-2267* (AD); Rockleigh Bushland Conservation Reserve, 22 Sep 2013, *D.E. Murfet 7615* (AD); Gosse-Ritchie Rd, 4.4 km N of South Coast Rd, Kangaroo Island, 8 Oct 1990, *B. Overton 1302* (AD); North Wilpena Creek, Wilpena Pound, Flinder’s Ranges, 22 Sep 1973, *A.J.A. Sikkes 788* (AD, CANB, PERTH); Manning Reserve near MacLaren Flat, 30 Oct 1963, *D.J.E. Whibley 1297* (AD); **New South Wales**: E side of Cuttabri Rd, 6.7 km due SW of Cuttabri, 18 Oct 2016, *D.E. Albrecht 14714 & R. Jobson* (CANB); Jingellic Nature Reserve, Black Ridge Firetrail, Coppabella Creek catchment, 3.75 km N of Jingellic, 11 Nov 2003, *I. Crawford 7922* (CANB); 30 m N of Walleroobie Rd, 200 m E of intersection with Ardlethan-Coolamon road, 27 Sep 2003, *D. Mallison 680* (CANB); S end of Narrandera Range, W of Mt Bogolong, ca. 3 km N of Newell Hwy and 11 km ENE of Narrandera, 1 Oct 1978, *T.B. Muir 6074* (MEL); **Victoria**: Dookie Agricultural College Reserve (hill 4 km S of Mt Major), 30 Sep 1992, *I. Crawford 1910*, *S. Mann & D. Robinson* (CANB); 1 km SW of Chewton and 4 km ESE of Castlemaine, 6 Oct 1981, *T.B. Muir 6742* (CANB, MEL); ca. 1 km N of Tallarook, 12 Oct 1978, *H.S. Meyer 23* (MEL); Paddles Quarry Block, 29 Sep 1988, *A. Paget 469* (MEL); Grampians National Park, Golton Track, ca. 50–100 m E of Mt Zero Rd, 25 Sep 2014, *R.W. Purdie 9711* (CANB, MEL); Chiltern-Mt Pilot National Park, Junction of Woolshed Rd with Masons Track, 13 Oct 2016, *N.G. Karunajeewa 1451* (MEL); **Tasmania**: Flinders Island, Strzeleckys [sic] Peak, [1844] *J. Milligan s.n.* (MEL); Pontville, [1874–1878] *W.W. Spicer s.n.* (MEL).

### 5. Levenhookia leptantha

Taxon classificationPlantaeAsteralesStylidiaceae

Benth., Fl. Austral. 4: 35. 1868

50DDB811-7D17-5736-AAD9-679C59A66ECA

Figs 1B , 2C–E , 4D

Leewenhoekia leptantha , orth. var.: F. von Mueller, Syst. Census Austral. Pl.: 86 (1882).

#### Type.

**Australia. Western Australia**: ‘*Drummond*, *n.* 128, 175, 282; Champion Bay and Murchison river, *Oldfield*; also a few specimens mixed in *Preiss’s n.* 2249 from Sussex district.’ Murchison River. Thicket south of Collallia [Colalya Creek], [1859–1860] *A.F. Oldfield 337* (lectotype, here designated: MEL 2257568; isolectotype: K 000060047); Champion Bay, Western Australia, *A.F. Oldfield s.n.* (syntype: MEL 2257566); Swan River, [?1843–1844] *J. Drummond [?3:]128* (syntype: K 000060046); Swan River, [1844–1847] *J. Drummond 4: 175* (syntypes: BM 001041269, CGE, G 00358743, G 00358744, K 000060044, K 000060048, MEL 2295753, P 00712439, W); Swan River, [1842] *J. Drummond 2: 282* (syntypes: BM 001041268, CGE, G 00358741, G 00358742, K 000060042, K 000060045, OXF, MEL 2295752, P 00712440, W); districtus Sussex, 20 Dec 1839, *L. Preiss 2249* (excluded syntype: MEL 2295747 *p.p.*), = *L. aestiva* [note: this gathering is also a syntype of *L. preissii*].

#### Description.

Annual herb 2–10 cm high. Glandular hairs 0.15–0.5 mm long. Stem reddish brown to dark red, simple (rarely once-branched at base), glandular-hairy. Leaves cauline, scattered, reddish to reddish brown; lamina succulent, usually lanceolate, ovate or elliptic, sometimes obovate or narrowly oblanceolate, 1–8 mm long including the petiole, 0.5–3 mm wide, subacute or acute, glandular-hairy abaxially and on the margins (sometimes sparsely so). Flowers usually in umbels, sometimes in short racemes, 1–30 per plant; bracts succulent, lanceolate, ovate or elliptic (sometimes narrowly so), 1–7 mm long, glandular-hairy like the leaves; pedicels 0.5–5 mm long, glandular-hairy. Hypanthium globose, ellipsoid or obovoid, 0.7–2 mm long, 0.6–1.7 mm wide, glandular-hairy. Calyx lobes ± equal, 0.8–2 mm long, acute, glandular-hairy. Corolla bright pink (rarely white) with a yellow (rarely creamy white) throat and dark pink or pink-red throat markings on each lobe (rarely absent), white abaxially; lobes evenly arranged or ± paired vertically, spreading to scarcely recurved, obovate, emarginate, retuse or incised, glabrous or with sparse glandular hairs abaxially near the base; anterior (lower) lobes ± equal in size or scarcely longer and broader than the posterior (upper) pair, 2–5.5 mm long, 2–4.5 mm wide; posterior lobes 1.8–5 mm long 1.8–4 mm wide, basally connate for 0.5–1 mm; tube creamy white or yellowish with pink longitudinal streaks, 3–9 mm long, 1.7–5 mm longer than the calyx lobes, sparsely glandular-hairy distally. Labellum ventral, 0.9–1.2 mm long including a short claw to ca. 0.2 mm long; hood yellow (rarely creamy white), usually with dark maroon markings near the cleft, sparsely glandular-hairy (mostly abaxially); appendage at the cleft apex yellow, ovate, elliptic or oblong, 0.2–0.5 mm long, minutely papillate, sometimes also with 1 or 2 glandular hairs; basal appendages yellow, rounded, 0.2–0.4 mm long, minutely papillate. Column sheath bright yellow (rarely pale greenish yellow), glabrous, with rounded anterior and lateral lobes 0.2–0.5 mm high, connate with the posterior corolla lobes forming a smooth, thickened pad, pendulous appendages absent. Column yellow, adnate to the anterior side of the corolla tube, 4.2–10 mm long with the top 0.7–1.1 mm free and slightly forward-arched when enclosed by the labellum, sparsely glandular-hairy distally on the anterior side; stigmatic lobes to ca. 0.6 mm long, the lower-most sharply upturned and developing while the column is hooded, the uppermost incurved and developing later. Mature capsules and seed not seen.

#### Diagnostic features.

*Levenhookia leptantha* has succulent floral bracts, a long (3–9 mm) corolla tube and comparatively short (0.8–2 mm long) calyx lobes, and minute glandular hairs at the tip of the column. Its corolla lobes are usually bright pink with a yellow base and the labellum has a morphologically distinct apical appendage that can be discerned on pressed material.

#### Phenology.

Flowering from late August to October.

#### Distribution.

*Levenhookia leptantha* is widespread in south-western Australia (Fig. 4C), although largely absent from the high rainfall zone, with most records occurring in the Geraldton Sandplains, Yalgoo, Avon Wheatbelt and Coolgardie bioregions. There are scattered occurrences in the Murchison and Mallee bioregions and an isolated record from the Swan Coastal Plain near Muchea.

#### Habitat.

*Levenhookia leptantha* grows in sand, sandy loam, sand over clay or clay loam on plains and gentle hill-slopes, often in association with granite outcropping, salt lakes, creek-lines or seasonally wet claypans. Associated vegetation is varied and includes open *Eucalyptus* woodland, mallee shrubland, tall shrubland or scrub with *Acacia*, *Allocasuarina*, *Melaleuca* or *Eremophila*, and halophytic dwarf shrubland. It is often found in more open areas of habitat growing with other ephemeral herbs.

#### Conservation status.

This widespread species is not currently considered to be at risk (IUCN 2012: Least Concern).

#### Etymology.

From the Greek *lepto*- (slender-) and *anthos* (flower): the flowers can appear narrow in pressed material due to their long and slender corolla tube.

#### Vernacular name.

Trumpet Stylewort (Erickson 1958).

#### Typification.

Bentham examined several gatherings when describing *L. leptantha*, including “a few specimens mixed in Preiss’s n. 2249” (a syntype of *L. preissii*). MEL 2295747 bears his corresponding annotation “These specimens seem rather to belong to *L. leptantha*, the lower ones to *L. preissii*”; however, I am at a loss to explain Bentham’s interpretation of this material. All individuals on this sheet appear referable to *L. aestiva* (refer to the typification section under *L. preissii*). A lectotype must therefore be selected from amongst the remaining material to fix the application of the name *L. leptantha*. MEL 2257568 has been selected since it is the best quality material with specific locality information that was viewed by Bentham. It bears a Botanical Museum of Melbourne label with the locality “Murchison River” as given by Mueller, an Oldfield label with the annotation “337. Fl. Pink. Moist places. Thicket south of Collallia” [Colalya Creek, which drains into the Murchison River east of Meekatharra] and a slip with “Gerald river Murchison” in Bentham’s hand. Bentham also retained a subset of this material at K.

#### Illustrations.

R. Erickson, Triggerplants 201, Pl. 57, No. 4 and 212, Pl. 59, Nos. 1–5. 1958 [only the free, distal portion of column depicted]; B.J. Grieve & W.E. Blackall, How to know W. Austral. wildfl. 4: 766, no. 5. 1982.

#### Selected specimens examined.

**Australia. Western Australia**: Bolgart, 40 km N of Perth, 29 Sep 1949, *R. Erickson s.n.* (PERTH); ca. 100 km E of Southern Cross, 26 Sep 1997, *B.A. Fuhrer 97/19* (PERTH); 29 miles [46.7 km] W of Mount Magnet, 11 Sep 1966, *A.S. George 79676* (PERTH); Gayon Station, Cue Road, Mullewa, 19.4 km NE of Courin Hill, 8 Oct 2004, *F. Hort*, *J. Hort & J. Shanks 2340* (PERTH); Emu Rock, Holland Track, 6 km SW from Hyden Norseman Rd, 22 Sep 2005, *R.W. Purdie 6097* (CANB, PERTH); Avon Loc, 19405, 1 mile [1.6 km] SW of Manmanning, 7 Oct 1988, *B.H. Smith 1101* (BRI, CANB, MEL); 850 m S along Wicherena Rd from Geraldton - Mt Magnet Rd, 12 Sep 1996, *J.A. Wege 194A & K.A. Shepherd* (PERTH); ca. 3.2 km E of Yellowdine on Great Eastern Highway, 13 Sep 2003, *J.A. Wege 895 & C. Wilkins* (PERTH); E of Canna Siding, 14 Sep 2011, *J.A. Wege 1828 & K.R. Thiele* (MEL, PERTH); ca. 3.1 km E of Great Northern Hwy on Goodlands Rd, NE of Jibberding Rocks, 12 Sep 2018, *J.A. Wege 2063* (MEL, PERTH); Bungabandi Creek on Eurardy Station, N of the Murchison River, 30 Aug 2003, *Wildflower Society of WA EURA 525* (PERTH).

### 6. Levenhookia pulcherrima

Taxon classificationPlantaeAsteralesStylidiaceae

Carlquist, Aliso 7(1): 62–64, figs 118, 119. 1969

111768CF-84C2-5F31-88A9-38C1CF0863FC

Figs 1E , 4F

#### Type.

**Australia. Western Australia**: Ongerup – Ravensthorpe Highway [precise locality withheld for conservation reasons], 8 Nov 1967, *S. Carlquist 4027* (holotype: RSA 0006328 image!; isotypes: AD 97031212 image!, AD 97133089 image!, B_10_0278639 image!, B_10_0278640 image!, BISH 1005114 image!, BRI-AQ0083605, CANB 195627, CHR 198044 image!, CHR 207972 image!, DAO 000457402 image!, E 00279220 image!, E 00279219 image!, GH 00033479 image!, K 000060049, K 000355298, L 0001769 image!, MEL 2295755, MEL 2295756, MICH 1192769 image!, MO-797445 image!, NCU 00000328 image!, OSC 0001733 image!, PERTH 01000284, PERTH 01000292, RM 0004403 image!, UC *n.v.*, US *n.v.*).

#### Description.

Annual herb 3–16 cm high. Glandular hairs 0.15–4 mm long. Stem dark red, often paler distally, simple or with porrect or ascending lateral branches, glandular-hairy. Leaves cauline, scattered, green adaxially, green or reddish abaxially; lamina oblanceolate to narrowly oblanceolate, lanceolate, ovate or elliptic, 4–22 mm long including the petiole, 1–5 mm wide, obtuse to subacute, sparsely glandular-hairy near the base on the margins and abaxial surface. Flowers in umbels, corymbs or a more elongated raceme, (1)3–ca. 100 per plant; bracts narrowly oblanceolate to almost linear, 4–20 mm long, glandular-hairy like the leaves; pedicels 0.5–4 mm long, glandular-hairy. Hypanthium depressed globose to globose or ellipsoid, 0.8–1.5 mm long, 0.7–1.3 mm wide, glandular-hairy. Calyx lobes subequal (with the anterior-most a little longer than the rest), 1.8–3.5 mm long, acute, sparsely to moderately glandular-hairy. Corolla bright to pale pink with a white throat (more rarely all white) and prominent dark pink markings on the posterior (upper) lobes; lobes paired vertically, spreading to scarcely recurved, obovate, incised or emarginate, usually with a few glandular hairs abaxially towards the base; anterior (lower) lobes slightly inwardly curved, a little longer and broader than the posterior (upper) lobes, 3.2–5 mm long, 2.5–3.6 mm wide; posterior lobes 3–4.5 mm long, 2.2–3.5 mm wide; tube 4.8–8 mm long, 2.5–5 mm longer than the calyx lobes, creamy white with pink-red longitudinal streaks, sparsely glandular-hairy distally. Labellum ventral, 1.3–2 mm long including a claw 0.2–0.4 mm long; hood purplish-red hood (drying dark red-maroon) with pinkish markings near the cleft, sparsely glandular-hairy abaxially, papillate adaxially along the margins of the cleft, cleft apex with a tuft of yellowish or whitish hairs; basal appendages creamy white, linear-subulate, 0.5–0.6 mm long. Column sheath creamy white, glabrous, with a narrowly triangular, obtuse, posterior lobe to 1 mm high and rounded anterior and lateral lobes 0.5–0.8 mm high, pendulous appendages absent. Column creamy-white, adnate to the anterior side of the corolla tube, 6.5–9 mm long with the top 1.5–2 mm free and angled toward the labellum, faintly constricted below the anthers, glabrous; stigmatic lobes to 1.2 mm long, apparently developing once the column has been exposed, the lowermost arching downwards, the uppermost straight to incurved. Capsule ovoid or globose, 2.5–4 mm long excluding calyx lobes. Seeds ca. 0.5 mm long, 0.3 mm wide.

#### Diagnostic features.

*Levenhookia pulcherrima* has an exserted corolla tube that is 4.8–8 mm long, incised or emarginate corolla lobes with dark pink marking near the base of the upper pair, and a shortly-stalked labellum with linear-subulate basal appendages and a small tuft of hairs at the tip.

#### Phenology.

Flowering from September to November; fruiting recorded from late October and November (and presumably extending into December).

#### Distribution.

*Levenhookia pulcherrima* is endemic to south-western Australia (Fig. 4E), where a small number of populations have been recorded from the central Avon Wheatbelt between Northam, Kellerberrin and Pingelly, and the Esperance Plains, Mallee and Coolgardie bioregions, from near Ravensthorpe to east of Forrestania.

#### Habitat.

This species grows in sand or loamy sand on floodplains, outwash hill-slopes or adjacent to granite outcropping. Associated vegetation includes *Acacia acuminata* woodland, *Allocasuarina* shrubland or scrub, mallee woodland or heath, or low open heath.

#### Conservation status.

This species is listed as Priority Three under Conservation Codes for Western Australian Flora (Western Australian Herbarium 1998–) (equivalent to IUCN (2012): Data Deficient). It remains poorly-known despite the discovery of seven new populations through assessment of herbarium collections and recent field surveys. Obtaining population data for this species is difficult since it is most abundant following fire (e.g. *J.A. Cochrane 6906 & B. Davis*, PERTH; *D.J. Edinger 935*, PERTH; *G.J. Keighery 466*, PERTH). A new population was surprisingly discovered in 2014 in a botanically well-surveyed nature reserve in the Avon Wheatbelt (*J. Borger & N. Moore MC 9-1*, PERTH) growing in unburnt habitat adjacent to experimental patch burns, suggesting that the smoke from these fires may have triggered germination. It is not known how long the soil seed bank remains viable and, as such, inappropriate fire regimes may represent a threat to this species.

#### Etymology.

From the Latin *pulcherrimus* (prettiest).

#### Vernacular name.

Beautiful Stylewort.

#### Notes.

Several collections of *L. pulcherrima* were previously misidentified as *L. leptantha*, a species with a similarly long corolla tube. Pressed material of *L. pulcherrima* can be separated from *L. leptantha* by its mostly longer calyx lobes (1.8–3.5 mm cf. 0.8–2 mm) and morphologically distinct labellum, which has linear-subulate basal appendages and an apical tuft of hairs (cf. with rounded basal appendages and a small, glabrous apical appendage). *Levenhookia pulcherrima* lacks the succulent leaves and bracts that characterise *L. leptantha* and the posterior side of its column sheath has a prominent triangular lobe (cf. sheath connate with the posterior corolla lobes to form a smooth, yellow pad).

A small, fast-moving, solitary native bee was observed gleaning pollen at *J.A. Wege 1937*.

#### Illustrations.

B.J. Grieve & W.E. Blackall, How to know W. Austral. wildfl. 4: 766, no. 4. 1982 [anterior corolla lobes mislabelled as “posterior petals” and vice versa].

#### Selected specimens examined.

**Australia. Western Australia**: [localities obfuscated for conservation reasons] W of Ravensthorpe, 11 Oct 1974, *S. Carlquist 6000* (PERTH); Phillips River, 27 Nov 2007, *J.A. Cochrane & B. Davis JAC 6906* (PERTH); Northam, Oct 1973, *G.J. Keighery 73.10/10* (PERTH); E of Lake King on Norseman Track, 27 Oct 1975, *G.J. Keighery 466* (PERTH); Phillips River, 27 Oct 1997, *B.J. Lepschi & B.A. Fuhrer BJL 3755* (CANB, MEL, PERTH); ENE of Lake King, 14 Nov 1979, *K.R. Newbey 6533* (PERTH); SE of Tammin, 19 Sep 2014, *J.A. Wege 1937* (MEL, PERTH); towards Ravensthorpe, 26 Oct 1968, *J.W. Wrigley s.n.* (CANB).

### 7. Levenhookia pauciflora

Taxon classificationPlantaeAsteralesStylidiaceae

Benth. in S.F.L. Endlicher, E. Fenzl, G. Bentham & H.W. Schott, Enum. Pl.: 74. 1837

50BE4731-0D03-55C8-A5DF-1B66013BA7F1

Figs 2G, H , 5B

Leeuwenhoekia pauciflora , orth. var.: A.P. De Candolle, Prodr. 7: 338. 1839.
Levenhookia stylidiodes F.Muell., Fragm. 6(43): 77. 1867, as Leeuwenhoekia. Type. Australia. Western Australia: “In Australia occidentali a sinu regis Georgii saltem usque ad montes Stirling’s Range. F.M.” King George’s Sound, Oct 1867, *F. Mueller s.n.* (syntypes: K 000060051, K 000060052, MEL 2257553, MEL 2257555, MEL 2257556, MEL 2257557, MEL 2257558, MEL 2257559, MEL 2257560); Albany, Oct 1867, *F. Mueller s.n.* (syntype: MEL 2257551); King George’s Sound, [no date] *F. Mueller s.n.* (probable syntype: MEL 2257557).
Leewenhoekia pauciflora , orth. var.: F. von Mueller, Syst. Census Austral. Pl.: 86. 1882.
Levenhookia stylidioides , orth. var.: B.D. Jackson, Index Kew. 2(3): 75. 1894.

#### Type.

**Australia. Western Australia**: King George’s Sound, [Jan 1834] *K. von Hügel s.n.* (holotype: W 0047174).

#### Description.

Annual herb 2–12 cm high. Glandular hairs 0.1–0.3 mm long. Stem dark red, sometimes paler reddish-brown distally, simple (rarely branched at base), glandular-hairy and papillate. Leaves cauline, scattered, green, sometimes tinged red; lamina broadly to narrowly ovate or occasionally reniform, 1.2–8 mm long including the petiole, 0.8–4 mm wide, obtuse or rounded, sparsely glandular-hairy abaxially near the base and on the margins, sometimes apparently glabrous. Flowers in short racemes or umbels, 1–15 per plant; bracts narrowly oblanceolate to oblanceolate or linear, 1–8 mm long, sparsely glandular-hairy abaxially and on the margins; pedicels 1–5(8) mm long, glandular-hairy. Hypanthium depressed globose or globose, 0.7–2.5 mm long, 0.8–2 mm wide, glandular-hairy. Calyx lobes equal or subequal (with the anterior pair scarcely longer than the rest and rarely connate at base), 1–3 mm long, obtuse to subacute, sparsely glandular-hairy. Corolla creamy white prominent red markings towards the base of the lobes at the tips of the upper lobes, abaxial surface with a fine red midvein, throat yellow; lobes paired vertically, sparsely glandular-hairy abaxially; anterior (lower) lobes narrowly oblanceolate, geniculate, longer and broader than the posterior pair, 3–7 mm long, 1.3–3.5 mm wide, apiculate or obtuse; posterior (upper) lobes lanceolate or elliptic, 2.5–4.5 mm long, 0.9–2.2 mm wide, acuminate, acute or apiculate; tube 2–3.5 mm long, ± equal to or up to ca. 1.2 mm longer than the calyx lobes, sparsely glandular-hairy distally. Labellum ventral, 1.5–3 mm long including a 0.2–0.6 mm claw; hood dark reddish maroon with yellow markings near the cleft, sparsely glandular-hairy abaxially, with a brush-tipped appendage 0.9–1.3 mm long at the cleft apex; basal appendages creamy white, linear to linear-subulate, 0.7–1.2 mm long. Column sheath yellow, lopsided, pendulous appendages absent; posterior side with 4 basally connate lobes 0.3–0.8 mm high, the lateral pair tipped with glandular hairs; anterior side adnate to the column, with 1 or 2 minute lobes visible on the anterior side of the column. Column creamy yellow, 3.2–6.5 mm long, adnate to the anterior side of the corolla (including the tube, lobes and column sheath) with the top 1.5–3 mm free and sharply angled towards the labellum, constricted below a dilated tip, glabrous; stigmatic lobes to ca. 1 mm long, developing once the column has been exposed, straight to incurved. Capsule globose or ovoid, 2–3 mm long excluding calyx lobes. Seeds 0.3–0.5 mm long, 0.2–0.4 mm wide.

#### Diagnostic features.

*Levenhookia pauciflora* has a stem with both glandular hairs and minute papillae, creamy white corolla lobes with a fine red midvein on the undersurface, upper corolla lobes with pointed tips, and a brush-tipped appendage at the apex of the labellum cleft. Its column and column sheath morphology are diagnostic but less readily viewed on pressed material.

#### Phenology.

Mostly flowering from September to early November (rarely in January as per type gathering); fruits have only been collected in October.

#### Distribution.

**Figure 5.**
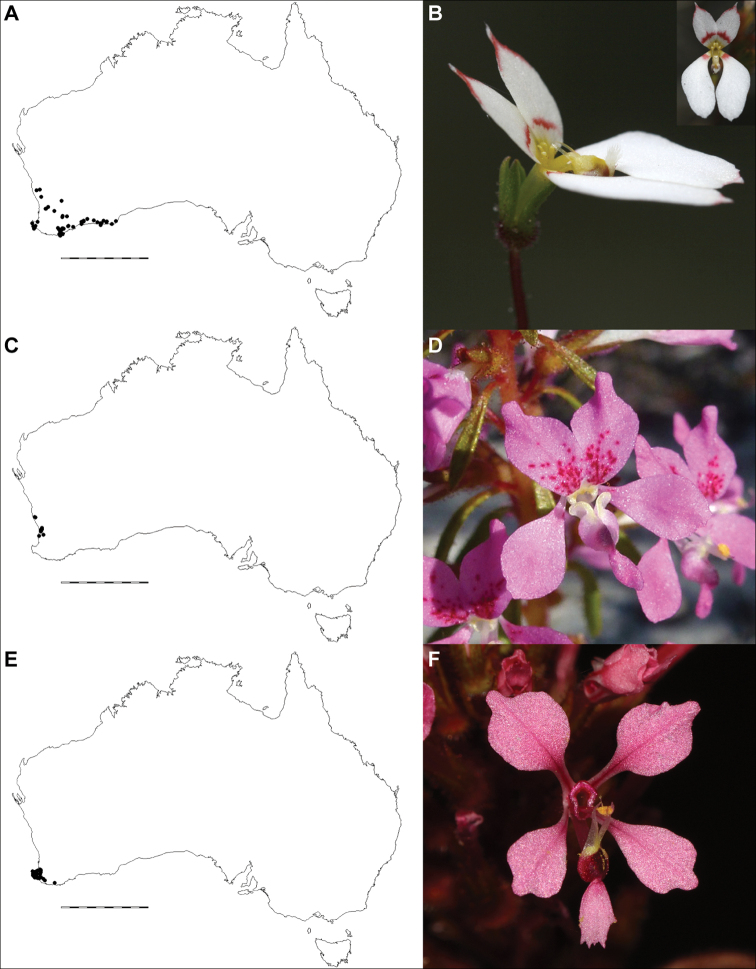
Comparative distributions and floral morphologies **A, B***Levenhookia pauciflora*, a Western Australian endemic with a brush-tipped labellum and distinctive corolla shape (*J.A. Wege 1071 & C. Wilkins*) **C, D***L. preissii*, a poorly-known Western Australian endemic with distinctive corolla makings, rounded basal labellum appendages and a white, lopsided column sheath (unvouchered, from NW of Cooljarloo) **E, F***L. aestiva*, a Western Australian endemic with oblong-subulate basal labellum appendages and a pink column sheath (*J.A. Wege 2090*). Photos by J.A. Wege (**B**), M. Matsuki (**D**) and R.W. Davis (**F**). Scale bar on maps 1000 km.

*Levenhookia pauciflora* is widespread in south-western Australia (Fig. 5A), extending from Badgingarra and Watheroo National Parks in the Geraldton Sandplains bioregion, south to Scott River National Park in the Warren bioregion, and east to Cape Arid National Park in the Esperance bioregion, with numerous records from the central Avon Wheatbelt and Mallee bioregions.

#### Habitat.

*Levenhookia pauciflora* is found on plains or hill-slopes in sand or clayey sand, sometimes with surface gravel or in association with granite outcropping. Associated vegetation is varied and includes heath, mallee shrubland, open *Eucalyptus* or *Corymbia* woodland, and scrub with emergent *Nuytsia* or *Banksia*.

#### Conservation status.

This is a widespread species that is not currently considered to be at risk (IUCN 2012: Least Concern).

#### Etymology.

From the Latin *paucus* (few) and -*florus* (-flowered).

#### Vernacular name.

Deceptive Stylewort (Erickson 1958). The corolla lobes of this species are reminiscent of some triggerplants including *Stylidium petiolare* Sond., with which it can co-occur.

#### Typification.

I annotated W 0047174 as a syntype during a visit to the Naturhistorisches Museum Wien in 2003 but have since failed to find any other duplicates of Hügel’s gathering. This sheet, which has been annotated by Bentham, is therefore regarded here as the holotype.

#### Illustrations.

R. Erickson, Triggerplants 201, Pl. 57, No. 6 and 212, Pl. 59, Nos. 19–23. 1958 [column and sheath morphology inaccurately depicted]; B.J. Grieve & W.E. Blackall, How to know W. Austral. wildfl. 4: 765, no. 2(1982); J. Wheeler, N. Marchant & M. Lewington, Fl. South West 2: 902 (2002).

#### Selected specimens examined.

**Australia. Western Australia**: Watheroo National Park, 12 Sep 1993, *K. Bremer & M. Gustafsson 110* (PERTH); 20 km E of Lake Grace on road to Newdegate, 14 Sep 1993, *K. Bremer & M. Gustafsson 122* (PERTH); S Stirling sandplain, S of Stirling Range, 16 Oct 1951, *R. Erickson s.n.* (PERTH); Mount Willying, N of Albany, 11 Oct 1969, *A.S. George 9694* (PERTH); Flint State Forest, Metro Rd, 6.1 km S from Brookton Hwy, 12 Oct 2009, *F. & J. Hort 3478* (PERTH); Plains S of Blackwood River, 24 Oct 1948, *R.D. Royce 2963* (PERTH); 14 km from Ravensthorpe towards Hopetoun, 12 Sep 1983, *J. Taylor 1712 & P. Ollerenshaw* (CANB); 3.7 km W along Cadda Rd from Brand Hwy, 23 Sep 1996, *J.A. Wege 209 & L. Cobb* (PERTH); 1.4 km E from NW boundary corner, Tarin Rock Nature Reserve, 21 Sep 1997, *J.A. Wege*, *R. Butcher & C. Wilkins JAW 358* (PERTH); 100 m E of Stockyard Rd on Merivale Rd, E of Esperance, 23 Sep 1997, *J.A. Wege 363*, *R. Butcher & C. Wilkins* (PERTH); 1.25 km E on Devil’s Creek Rd from Gairdner River Crossing, NW of Bremer Bay, 29 Sep 1997, *J.A. Wege 380*, *R. Butcher & C. Wilkins* (PERTH); ca. 1 km N of park boundary on Chester Pass Rd, Stirling Range National Park, 30 Oct 2003, *J.A. Wege 1071 & C. Wilkins* (PERTH); 8 miles [12.9 km] W of Israelite Bay, 1 Oct 1968, *P.G. Wilson 8148* (AD, CANB, MEL, PERTH).

### 8. Levenhookia preissii

Taxon classificationPlantaeAsteralesStylidiaceae

(Sond.) F.Muell., Fragm. 4(27): 94, 1864, as Leewenhoekia

1B79092C-310F-5833-BD01-4217EEE0EA8F

Fig. 5D

Coleostylis preissii Sond., in J.G.C. Lehmann, Pl. Preiss. 1(3): 391. 1845.
Leewenhoekia preissii , orth. var.: F. von Mueller, Syst. Census Austral. Pl.: 86. 1882.

#### Type.

**Australia. Western Australia**: Swan River, [1841] *J. Drummond 1: 515* (lectotype, here designated: BM 000984007; isolectotypes: G 00342974 image!, G 00342988 image!, K 000060074, K 000060076, MEL 2295745, MEL 2295746, OXF, P 00712441 image!, P 00712444 image!, P 00712443 image!, W [2 sheets]); In arenosis cis oppidulum Guildford, 12 Jan 1840, *L. Preiss 2250* (syntypes: G 00358747 image!, G 00358748 image!, K 000060078 [as Preiss 842], LD 1730899 image!, MEL 2295748, MEL 2295750B [top individual], P 00712442 image!, TCD [as Preiss 842], W); In arenosis districtus Sussex, 20 Dec 1839, *L. Preiss 2249* (syntypes: FI 012788!, G 00358745 image!, G 00358746 image!, K 000060077 [as Preiss 895], LD 1753988 image!, MEL 2295747, MEL 2295749A [2 upper individuals], MEL 2295750A [lower 3 individuals], P 00712441 image!, TCD [as Preiss 895], W [3 sheets]), = *L. aestiva* Wege.

#### Description.

Annual herb 6–16 cm high. Glandular hairs somewhat viscid, 0.1–0.5 mm long. Stem pale greenish brown to reddish brown, simple or branched to varying degrees with porrect or ascending lateral branches, glandular-hairy. Leaves cauline, scattered, green or green with a red tinge, glandular-hairy on the margins and abaxially, with a few hairs adaxially (mostly towards the base); lamina narrowly oblanceolate to oblanceolate or narrowly lanceolate, 6–22 mm long including the petiole, 0.9–3 mm wide, acute to subacute with a blunt tip. Flowers in racemes (inflorescence corymbose in few-flowered individuals), ca. 10–200^+^ per plant; bracts narrowly oblanceolate, narrowly lanceolate or ± linear, 1.5–12 mm long, glandular-hairy like the leaves; pedicels 0.5–6 mm long, glandular-hairy. Hypanthium depressed globose to globose, ellipsoid or ovoid, 0.5–1.5 mm long, 0.4–1.5 mm wide, glandular-hairy. Calyx lobes subequal (with the anterior pair 0.5–1 mm longer than the rest and sometimes connate basally), 1.3–3 mm long, acute, glandular-hairy. Corolla pink with dark pink speckles mostly confined to the posterior (upper) lobes, paler abaxially; lobes ± paired vertically or sometimes with the lower pair spreading, ca. 3–4.5 mm long, ca. 1.5–2.5 mm wide, sparsely glandular-hairy abaxially towards the base; anterior lobes elliptic or obovate with a slender claw, ± equal in length to the posterior pair, rounded or subacute; posterior lobes obovate (with a broad but short claw?), bluntly pointed, sometimes gently recurved; tube white, 2–4.5 mm long, exserted 0.5–2 mm beyond the calyx lobes, sparsely glandular-hairy distally. Labellum ventral, ca. 3–4.2 mm long including a 1.2–1.7 mm long claw; hood pink with dark purplish (drying red-maroon) markings, minutely papillate, with a few glandular hairs abaxially; basal appendages yellow or white, ± elliptic, 0.4–0.7 mm long, rounded, minutely papillate; appendage at the cleft apex pink, elliptic to narrowly obovate, 0.7–1.5 mm long, 0.4–0.6 mm wide, obtuse, glabrous. Column sheath white, glabrous, lopsided, ca. 0.5–0.7 mm high on the anterior side, connate with the posterior corolla lobes, with a thickened rim bearing 3 pendulous appendages on the inside. Column white basally, pinkish distally, free, gently forward-arched when enclosed by the labellum, slender but slightly thickened distally, 4.5–7.2 mm long, glabrous; stigmatic lobes to 1.2 mm long, incurved, apparently maturing subsequent to pollen release. Capsule ovoid, ca. 2.5–3 mm long excluding calyx lobes. Seeds 0.4–0.5 mm long, 0.2–0.3 mm wide.

#### Diagnostic features.

*Levenhookia preissii* has a long (2–4.5 mm) corolla tube that is exerted beyond the calyx lobes, pink corolla lobes with speckled markings, a long labellum (ca. 3–4.2 mm) with rounded basal appendages and a prominent (0.7–1.5 mm long) apical appendage, and a 4.5–7.2 mm long column subtended by a lopsided column sheath. Its racemes are usually quite elongated (especially in floriferous individuals).

#### Phenology.

Flowering from late October to January; fruiting in December and January.

#### Distribution.

*Levenhookia preissii* is endemic to south-western Australia (Fig. 5C), where it is restricted to the Swan Coastal Plain bioregion. It is currently known from north-west of Cataby and the Perth metropolitan area, although historical records indicate a distribution that extends south to the Pinjarra area.

#### Habitat.

This species grows in sand in seasonally-wet habitats or near watercourses, swamps and minor drainage channels in heath or tall shrubland. Associated species include *Banksia telmatiaea*, *Beaufortia squarrosa*, *Hypocalymma angustifolium*, *Melaleuca viminea*, *M. seriata*, *Regelia ciliata* and *Verticordia densiflora*.

#### Conservation status.

*Levenhookia preissii* was recently listed as Priority One under Conservation Codes for Western Australian Flora (Western Australian Herbarium 1998–; equivalent to IUCN (2012): Data Deficient). It is currently known from two bushland fragments in Perth and from an area north-west of Cataby that is subject to mining. Few plants have been noted with the exception of *B.J. Keighery 2546*, which was collected the year following a summer fire. The optimal time for survey and conservation assessment is during peak flowering (between mid-November and mid-December), following a summer or autumn fire.

#### Etymology.

Honours Johann August Ludwig Preiss (1811–1883), who collected extensively in south-western Australia between 4 December 1838 and 8 January 1842 (George 2009). These collections formed the basis for Lehmann’s *PlantaePreissianae*, a landmark work on Western Australian botany in which a suite of Stylidiaceae taxa were described (Sonder 1845).

#### Vernacular name.

Preiss’s Stylewort (Erickson 1958).

#### Typification.

Sonder based his description of *Coleostylis preissii* on three separate gatherings of which *Preiss 2250* (from the Perth suburb of Guildford) and *Drummond 515* (‘Swan River’) are comparable; however, *Preiss 2249* (from the more southerly ‘Sussex District’, i.e. between Capel and Toby’s Inlet: see Marchant 1990) represents a distinct species. Lectotypification is necessary to fix the application of the name *L. preissii*.

Sonder viewed material of both Preiss gatherings at LD and in his personal herbarium, which is now at MEL. The LD material is fragmentary, comprising one or two inflorescence portions. The Preiss material at MEL is more complete but difficult to interpret—there are few readily visible flowers, labelling is ambiguous and one of the sheets (MEL 2295750) contains material from both gatherings. This sheet includes four individuals, several floral dissections and Sonder’s sketches and notes (which indicate that he observed differences in labellum size and appendage morphology between the two gatherings). The uppermost individual belongs to *Preiss 2250*, while the remaining three individuals appear to match *Preiss 2249*. I have not been able to confidently assign all fragments contained in the attached packet, but I have separated several that seem to be referable to *Preiss 2250*, including dissected flowers. I have been unable to find a dissection for *Preiss 2249* despite Sonder’s floral sketches.

Given the difficulties associated with interpreting the Preiss material at MEL, the fragmentary nature of the duplicates at LD and, indeed, the generally poor quality of the material, I have made the pragmatic decision to lectotypify on the Drummond gathering despite its lack of precise locality. Based on our present day understanding of the species and Drummond’s collecting trips (see George 2009), it is likely to have been collected from the Perth region. BM 000984007 is designated as the lectotype: it comprises four, mostly complete individuals with ample floral material, a small packet with some fragments and a pink annotation slip with the name ‘Coleostylis racemiflora Sond.’ in Sonder’s hand (an earlier manuscript name that he also wrote on a subset of material at LD and MEL, but subsequently amended). MEL 2295746, from Sonder’s Herbarium, is interpreted as a likely duplicate given that there is an original label with the collector and number (although a photocopy of the *Preiss 2249* and *2250* label from MEL 2295750 is also affixed to this sheet). MEL 2295745 is also a duplicate—it has a label in Mueller’s hand and was seen by Bentham for *Flora Australiensis*, although it does not bear Sonder’s script (although is databased as belonging to Sonder’s Herbarium).

#### Notes.

There are several morphological features that support a narrower circumscription of *L. preissii* and the recognition of *L. aestiva* as a distinct species (refer to the comparative notes provided under *L. aestiva*). The two species are geographically separated: historical records of *L. preissii* extend as far south as the Serpentine and Murray River area near Pinjarra, while the northern-most records of *L. aestiva* are from the Preston River area east of Bunbury.

#### Illustrations.

L. Diels & E. Pritzel, Bot. Jahrb. Syst. 35: 598, fig. 67 D–G (1905) [reproduced by J. Mildbraed in H.G.A. Engler, Pflanzenr. 35: 29, fig. 10D–G]; R. Erickson, Triggerplants 201, Pl. 57, No. 7 and 212, Pl. 59, Nos. 11–18 [column sheath not clearly depicted].

#### Specimens examined.

**Australia. Western Australia**: [precise localities obfuscated for conservation reasons] Guildford, 5 Jan 1951, *Anonymous s.n.* (PERTH); Bayswater, Dec 1901, *C. Andrews s.n.* (PERTH); Bullsbrook area, 28 Dec 1971, *N.T. Burbidge 7960* (CANB); Bayswater, Dec 1900, *Dr Diels & Pritzel 409* (PERTH); Guildford, Jan 1954, *R. Erickson s.n.* (PERTH); Cannington, Oct 1898, *R. Helms s.n.* (PERTH); Perth Airport, 20 Oct 1994, *B.J. Keighery 2546* (PERTH); NW of Cooljarloo, 1 Dec 2014, *M. Matsuki 197A* (PERTH); Cannington, 14 Dec 1898, *A. Morrison s.n.* (BRI, CANB, PERTH); Bayswater, 9 Jan 1899, *A. Morrison s.n.* (PERTH); Serpentine River, 1 Dec 1877, *F. von Mueller s.n.* (MEL); Murray River district, Dec 1900, *E. Pritzel 128* (PERTH); Bayswater, 26 Dec 1924, *O.H. Sargent s.n.* (PERTH); [SE of Cervantes], 24 Nov 2005, *G. Woodman Opp 5* (PERTH); Leeming, 17 Dec 2011, *J.E. Wajon JEW 2533* (PERTH).

### 9. Levenhookia aestiva

Taxon classificationPlantaeAsteralesStylidiaceae

Wege sp. nov.

7E81FDCD-E7E4-515B-A209-3F8F92951170

urn:lsid:ipni.org:names:77209908-1

Figs 2B , 5F , 6.

Levenhookia sp. Whicher Range (J.A. Wege 2090), Western Australian Herbarium, in *FloraBase*, https://florabase.dpaw.wa.gov.au/ [accessed 6 March 2020].

#### Diagnosis.

*Levenhookia aestiva* can be identified by its long (5.5–8 mm) corolla tube, long (7.5–11 mm) column, entire column sheath with 3 pendulous appendages, and long labellum (4.5–6.5 mm) with a prominent apical appendage and oblong-subulate basal appendages.

#### Type.

**Australia. Western Australia**: 1.1 km E on Sabina East Road from Sues Road, Whicher National Park, 18 Dec 2018, *J.A. Wege 2090* (holo: PERTH 09082654; iso: AD, CANB, K, MEL, NSW).

#### Description.

Annual herb 4–15 cm high. Glandular hairs somewhat viscid, 0.2–0.6 mm long. Stem dark red or brownish red, simple or branched to varying degrees with porrect or ascending lateral branches, glandular-hairy. Leaves cauline, scattered, green or red, glandular-hairy on the margins and abaxially, with a few hairs adaxially (mostly towards the base); lamina narrowly oblanceolate to oblanceolate, elliptic or ovate, 3–25 mm long including the petiole, 1–5.5 mm wide, acute to subacute with a blunt tip. Flowers in racemes (inflorescence corymbose in few-flowered individuals), 1–ca. 300-flowered; bracts narrowly oblanceolate to oblanceolate, narrowly lanceolate or ± linear, 2–22 mm long, glandular-hairy like the leaves; pedicels 1–6 mm long, glandular-hairy. Hypanthium globose, obovoid or depressed-obovoid, 0.7–1 mm long, 0.8–1.5 mm wide, glandular-hairy. Calyx lobes subequal (with the anterior pair scarcely longer than the rest and rarely connate basally), 2.5–4.2 mm long, acute, glandular-hairy. Corolla pink, often with a dark pink midvein and with white or pale pink margins near the base of the lobes; lobes evenly arranged tending vertically-paired, elliptic or obovate with a slender claw, usually slightly recurved, sparsely glandular-hairy abaxially towards the base and along the midvein; anterior (lower) lobes slightly narrower than the posterior pair, 4.5–6.5 mm long, 2.2–3.2 mm wide, bluntly pointed or rounded; posterior (upper) lobes 4.5–6 mm long, 2.8–3.5 mm wide, bluntly pointed; tube whitish with pink longitudinal stripes distally, 5.5–8 mm long, exserted 1.5–4.5 mm beyond the calyx, sparsely glandular-hairy distally. Labellum ventral, 4.5–6.5 mm long including a 1.5–2.5 mm long claw; hood pink with dark red-purple markings adaxially, minutely papillate, sometimes with a few glandular hairs abaxially; basal appendages creamy white, pale yellow near the base, oblong-subulate, 1–2.5 mm long, acute or obtuse, sometimes papillate; appendage at the cleft apex pink, elliptic to obovate, 1.5–2 mm long, 0.8–1.4 mm wide, usually with an irregularly incised apex (rarely obtuse), glabrous. Column sheath deep pink, glabrous, 0.5–1 mm high, with an entire, thickened rim bearing 3 pendulous appendages on the inside. Column white basally, pinkish distally, free, forward-arched when enclosed by the labellum, slender but slightly thickened distally, 7.5–11 mm long, glabrous; stigmatic lobes to 1.3 mm long, incurved, apparently maturing subsequent to pollen release. Capsule depressed obovoid, 1.5–3 mm long excluding calyx lobes. Seeds 0.4–0.5 mm long, 0.2–0.3 mm wide.

**Figure 6.**
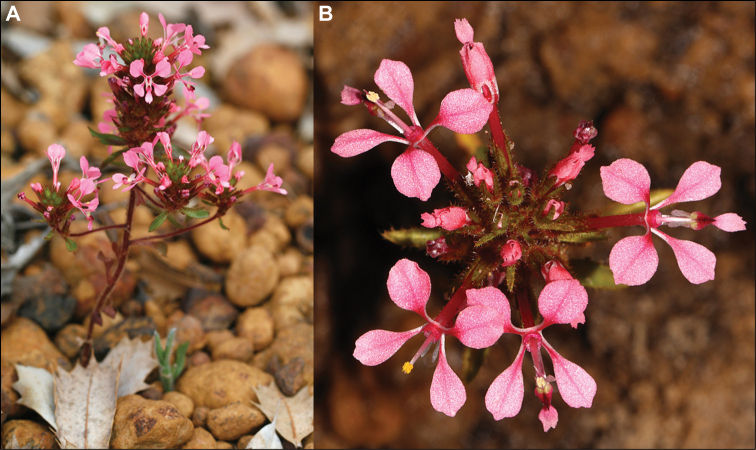
*Levenhookia aestiva* (*J.A. Wege 2090*) **A** annual habit showing the ascending lateral branches; **B** inflorescence as viewed from above, showing flowers with a long corolla tube and column and a pink, entire column sheath. Photos by J.A. Wege (**A**) and R.W. Davis (**B**).

#### Phenology.

Mostly flowering from mid-November to early February, with flowering extending into March and April in swampy habitats on the south coast; mostly fruiting from mid-December to February.

#### Distribution.

Most records of *L. aestiva* are from the south-west corner of Western Australia (Fig. 5E) between Bunbury, Pemberton, Augusta and Yallingup in the Warren, Jarrah Forest and Swan Coastal Plain bioregions. There is an outlying, but morphologically comparable record near Denmark.

#### Habitat.

*Levenhookia aestiva* grows in sandy soils near swamps and on low lying flats, or in sandy loam with lateritic gravel in more upland habitats. It is commonly recorded from post-fire habitats and disturbed roadsides or firebreaks. Associated vegetation includes open *Eucalyptus marginata*, *Corymbia calophylla* or *C. haematoxylon* woodland with *Kingia australis* and *Xanthorrhoea preissii*, and low Myrtaceous shrubland. In lateritic habitats, it may grow in sympatry with *Stylidium lateriticola* Kenneally, a species with similarly bright pink, summer-blooming flowers.

#### Conservation status.

Despite its reasonably narrow geographic range, *L. aestiva* is locally common at numerous sites within the conservation estate (especially following a disturbance) and is not currently considered to be at risk (IUCN (2012): Least Concern).

#### Etymology.

From the Latin *aestivus* (of summer), in reference to its flowering time.

#### Vernacular name.

Summer Stylewort.

#### Notes.

Specimens of *L. aestiva* have previously been placed under a broadly defined *L. preissii* (Sonder 1845, Mildbraed 1908, Erickson 1958, Wheeler 2002, Lowrie and Conran 2011); however, there are several features that support its recognition as a distinct species. Corolla tube length (5.5–8 mm cf. 2–4.5 mm in *L. preissii*) and column length (7.5–11 mm cf. 4.5–7.2 mm in *L. preissii*) are taxonomically informative and readily observed on pressed material. *Levenhookia aestiva* also has a longer labellum (4.5–6.5 mm cf. 3–4 mm) with a larger apical appendage (1.5–2 mm × 0.8–1.4 mm cf. 0.7–1.5 × 0.4–0.6 mm) and longer basal appendages (1–2.5 mm cf. 0.4–0.7 mm long). Its corolla lobes lack the speckled markings that appear characteristic of *L. preissii* (compare Fig. 5D, F) and its nectar sheath is morphologically distinct. Capsule shape may also be taxonomically informative (depressed obovoid in *L. aestiva* cf. ovoid in *L. preissii*), although few mature capsules of the latter species have been viewed. The two species are not known to overlap in distribution (refer to the notes under *L. preissii*).

#### Selected specimens examined.

**Australia. Western Australia**: 6.7 km N on Black Point Rd from Wapet Track, 30 Jan 1997, *E. Bennett & B. Evans P 13.1* (PERTH); Creek View Rd, Reserve 12492, Quindalup, 10 Dec 2003, *D. Carter 661* (PERTH); 32 km from Pemberton along road to Nannup, 21 Jan 1979, *M.D. Crisp 5350* (CANB, PERTH); 15 km E of Karridale, 15 Jan 1996, *R. Davis RD 443* (PERTH); Margaret River crossing on Rapid Rd, Whicher Range, 16 Jan 1986, *A.H. Burbidge 3966* (PERTH); Denmark Shire, Nutcracker Rd, 1 km E from junction with Stan Rd, 2 Jan 1999, *B.G. Hammersley 2150* (PERTH); 1 km along Rapids Rd from Canebreak Rd, Whicher Range, ca. 20 km S of Busselton, 13 Jan 1986, *G.J. Keighery 8046* (CANB, PERTH); Forest Grove Block, 10 km S of Margaret River, 10 Dec 1990, *G.J. Keighery 13750* (PERTH); Capel Nature Reserve, 13 Dec 1994, *G.J. Keighery 13260* (PERTH); Ambergate Regional Park, 13 km SSW of Busselton, 14 Nov 1994, *G.J. Keighery 15146* (PERTH); Joshua Brook Rd, Boyanup Forest Block, 15 Jan 1997, *G.J. Keighery 15067* (PERTH); 7.25 km SW along Sabina Rd from Vasse Hwy, S of Busselton, 29 Nov 1995, *J.A. Wege 154 & P. French* (PERTH); Sues Bridge camping area on the Blackwood River, 50–100 m W of Sues Rd, SE of Busselton, 29 Jan 2009, *J.A. Wege 1590* (PERTH); 6.1 km S of Governor Broome Rd on Milyeannup Coast Rd, N of Scott River, E of Augusta, 30 Jan 2009, *J.A. Wege 1592* (PERTH).

### 10. Levenhookia stipitata

Taxon classificationPlantaeAsteralesStylidiaceae

(Benth.) F.Muell. ex Benth., Fl. Austral. 4: 36. 1868

32CF3AD7-8F90-54E5-8921-BB3752C8136F

Figs 2A , 7B

Stylidium stipitatum Benth., in S.F.L. Endlicher, E. Fenzl, G. Bentham & H.W. Schott, *Enum. Pl.*: 72. 1837.
Coleostylis umbellata Sond. in J.G.C. Lehmann, Pl. Preiss. 1(3): 391. 1845, *nom. illeg.*[Stylidium stipitatum cited in synonymy]
Leewenhoekia stipitata (Benth.) F.Muell., Fragm. 4(27): 94. 1864, *nom. inval.*, *nom. prov.*
Leewenhoekia stipitata , orth. var.: F. von Mueller, Syst. Census Austral. Pl.: 86. 1882.

#### Type.

**Australia. Western Australia**: Swan River, [1833] *K. von Hügel s.n.* (lectotype, here designated: W 0047173, all *Levenhookia* material [i.e. excluding the individual of *Centrolepis*]).

#### Description.

Annual herb 2–18 cm high. Glandular hairs 0.1–0.5 mm long. Stem dark red to reddish brown, sometimes paler or greenish distally, simple or branched to varying degrees with porrect or ascending lateral branches, glandular-hairy. Leaves cauline, scattered, green (occasionally tinged red) or red-purple, lamina narrowly oblanceolate to oblanceolate, elliptic or ovate, 2.5–12 mm long including the petiole, 0.5–3(–4.5) mm wide, subacute to acute or obtuse, glandular-hairy on the abaxial surface and margins. Flowers in umbels, corymbs or short racemes, 1–ca. 150 per plant; bracts narrowly oblanceolate to oblanceolate, elliptic or linear, 2–10 mm long, glandular-hairy like the leaves; pedicels 3–18 mm long, glandular-hairy. Hypanthium depressed globose, globose or ellipsoid, 0.7–1.5 mm long, 0.7–1.8 mm wide, glandular-hairy. Calyx lobes ± equal or with the anterior pair scarcely longer than the rest, 1.3–2.3 mm long, acute, moderately to sparsely glandular-hairy. Corolla bright to pale pink (rarely white) with a white throat, sometimes with two, elongated red-pink markings towards the base of each lobe near the margins; lobes evenly arranged or with the upper (posterior) ones ± paired vertically, sometimes weakly recurved, obovate or elliptic with a slender claw, rounded, scarcely apiculate or bluntly pointed, glabrous or sometimes with sparse glandular hairs abaxially; anterior lobes equal to or slightly shorter and narrower than the posterior pair, 2.5–4.5 mm long, 1.6–2.8 mm wide; posterior lobes 2.7–4.5 mm long, 1.7–3.3 mm wide; tube creamy white, 0.2–0.5 mm long, obscured by the calyx lobes, glabrous. Labellum ventral, 2–3.5 mm long including a 0.9–1.5 mm long claw; hood yellow or whitish with dark red-maroon markings, sparsely glandular-hairy abaxially, scarcely papillate adaxially along the margins, papillate abaxially with a short, blunt, dark pink-red or sometimes yellow appendage at the cleft apex; basal appendages yellowish or white, rounded, 0.4–0.6 mm long. Column sheath white or yellowish, glabrous, 1.3–2.5 mm high on the posterior side with a thickened rim and broad anterior cleft, 3 pendulous appendages present on the inner surface towards the throat. Column creamy white, free, erect, slightly thickened distally, 2–4 mm long; stigmatic lobes to ca. 1 mm long, incurved, the lower-most developing while the column is hooded, the uppermost developing later. Capsule globose or ovoid, 1–2.3 mm long excluding calyx lobes. Seeds 0.4–0.5 mm long, 0.25–0.3 mm wide.

#### Diagnostic features.

*Levenhookia stipitata* has glandular-hairy stems, bracts and calyx lobes, long pedicels (3–18 mm), a short corolla tube (obscured by the calyx lobes), a small, blunt, papillate appendage at the tip of the labellum, and a prominent column sheath (more than half the length of column). The corolla lobes may have a pair of elongated markings near the base (see notes below).

#### Phenology.

Flowering from September to December; fruits have been collected from late October to December.

#### Distribution.

**Figure 7.**
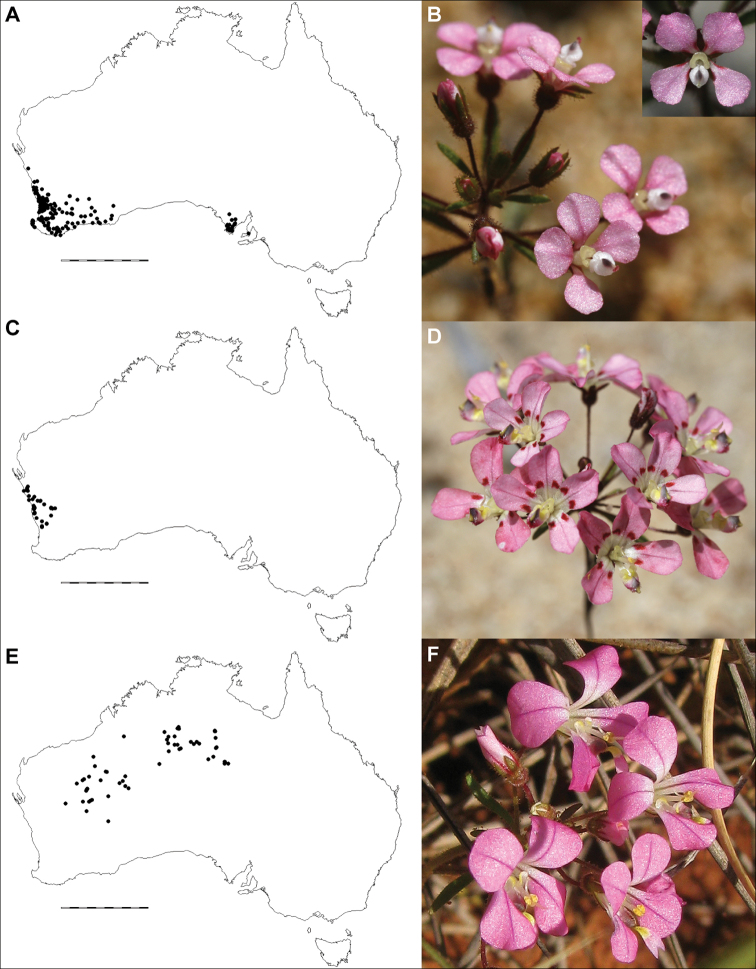
Comparative distributions and floral morphologies **A, B***L. stipitata*, with a disjunct distribution in Western Australia and South Australia and flowers on long pedicels and with a prominent column sheath (*J.A. Wege 1874*). Inset showing coloured markings at the base of the corolla lobes that have been recorded for many populations (*J.A. Wege 1873*) **C, D***L. octomaculata*, a Western Australian endemic with prominent corolla markings and a densely papillate labellum (*J.A. Wege 2074*) **E, F***L. chippendalei*, widespread in arid areas of Western Australia and the Northern Territory, with the incised apical labellum appendage visible in the lower right flower (*N. Gibson 6559*, *S. van Leeuwen*, *M.A. Langley & K. Brown*). Photos by J.A. Wege (**B, D**) and K. Brown (**F**). Scale bar on maps 1000 km.

This species is widespread in Western Australia (Fig. 7A), occurring in all bioregions within the South-West Province as well as the adjacent Yalgoo and Coolgardie bioregions. It has a more restricted distribution in South Australia where it is mostly confined to the Eyre Peninsula between Pinkawillinie Conservation Park and Point Boston, with an outlying record from the central Yorke Peninsula.

#### Habitat.

*Levenhookia stipitata* grows in sand, sandy loam or clayey sand over limestone or laterite, more rarely in association with granite outcropping, usually on plains and hill-slopes but sometimes in depressions or seasonally damp habitats. Associated vegetation is varied and includes heathland, woodland, *Eucalyptus marginata* and *Corymbia calophylla* forest, *Banksia*, *E. wandoo* or *E. cladocalyx* woodland, open mallee woodland or shrubland, and *Acacia* or *Melaleuca* tall shrubland.

#### Conservation status.

This common species is not considered to be under threat (IUCN (2012): Least Concern); however, in South Australia, it is listed as Rare (Schedule 9) under the *National Parks and Wildlife Act 1972* (Government of South Australia 2018).

#### Etymology.

From the Latin *stipitatus* (stipitate, provided with a stipe or little stalk), presumably with reference to the corolla segments.

#### Vernacular name.

Common Stylewort (Erickson 1958).

#### Typification.

The only Hügel specimen that is known is held at Naturhistorisches Museum Wien (W 0047173) and bears annotations by Bentham (as ‘*Stylidium stipitatum*’), Sonder (as ‘*Coleostylis umbellulata*’) and Mildbraed (as ‘*Levenhookia stipitata*’). The collection is mixed, comprising four individuals of *L. stipitata* (and associated fragments in the attached packets) and a single individual of *Centrolepis aristata* (R.Br.) Poir. (Centrolepidaceae) mounted with the *Levenhookia* in the centre of the sheet. All *Levenhookia* material on this sheet is designated herein as the lectotype.

#### Notes.

Corolla markings are often absent in *L. stipitata*; however, two discrete markings at the base of each lobe can be consistently present in populations in Western Australia and South Australia (e.g. *D.E. Murfet 2270 & R.L. Taplin* (AD), *D.E. Murfet 4494 & A. Lowrie* (AD), *J.A. Wege 1562 & B.P. Miller* (PERTH), *J.A. Wege 1873* (PERTH: Fig. 7B, inset), *J.A. Wege* 2068 (PERTH)) or variably present within a population (e.g. *J.A. Wege 777*, *J.A. Wege 2077*). A comparison with *L. octomaculata*, an allied species named for its corolla markings, is provided under the notes for that species.

#### Illustrations.

L. Diels & E. Pritzel, Bot. Jahrb. Syst. 35: 598, fig. 67 A–C. 1905 [reproduced by J. Mildbraed in H.G.A. Engler, Pflanzenr. 35: 29, fig. 10A–C. 1908]; R. Erickson, Triggerplants 201, Pl. 57, No. 2 and 208, Pl. 58, Nos. 8–14. 1958; B.J. Grieve & W.E. Blackall, How to know W. Austral. wildfl. 4: 766, no. 6. 1982; J. Wheeler, N. Marchant & M. Lewington, Fl. South West 2: 903. 2002.

#### Selected specimens examined.

**Australia. Western Australia**: Mt Merivale, 20 km E of Esperance, 25 Oct 1995, *B. Archer 168* (MEL); Brixton Street Wetlands, Kenwick, 10 Nov 2011, *K.L. Brown 888 & G. Paczkowska* (PERTH); Darling Range escarpment, W of Walyunga Reserve, 17 Dec 1971, *N.T. Burbidge 7882* (CANB); Dryandra State Forest, NE of Congelin, at the sigma bend of Patonga Rd, 24 Oct 1991, *W. Greuter 23183* (PERTH); Sheepwash State Forest, E boundary at ‘The Pass’, 13 Dec 1998, *B.G. Hammersley 2137* (PERTH); Reserve 23229/1255, Brookton Hwy, Armadale, 5 km SE of Kinsella Rd, 11 Dec 2004, *F. Hort*, *J. Hort & B. Hort 2451* (PERTH); Wongamine Nature Reserve, ca. 13 km NE of Toodyay, 7 Oct 1995, *T.R. Lally & B.J. Lepschi 775* (PERTH); Sukey Hill, 3.5 km E of Cranbrook, 13 Nov 1995, *T.R. Lally & B.J. Lepschi 880* (PERTH); 1.85 km along Park Rd from Great Eastern Hwy, John Forrest National Park, 14 Nov 1995, *J.A. Wege 120* (PERTH); 20 m NE along gravel track off Del Park Rd, 6.7 km from South Western Hwy, 29 Nov 1995, *J.A. Wege 156 & P. French* (PERTH); ca. 900 m from Eagle Bay settlement on Meelup Beach Rd, 8 Nov 2002, *J.A. Wege 777* (PERTH); 1.6 km E on Cadda Rd from Brand Hwy, Badgingarra National Park, *J.A. Wege 1688 & W.S. Armbruster*, 20 Oct 2009 (PERTH); ca. 1.7 km on Canning Road from Pickering Brook Road, Korung National Park, 12 Nov 2003, *J.A. Wege 1100* (PERTH); Qualen Rd, just W of Kent Rd, Wandoo National Park, 29 Nov 2008, *J.A. Wege & B.P. Miller JAW 1562* (PERTH); 6.4 km W of Brand Hwy on Bibby Rd, SW of Badgingarra, 20 Oct 2011, *J.A. Wege 1873* (PERTH); 4.7 km E of Brand Hwy on Wanamal West Rd, Boonanarring Nature Reserve, 20 Oct 2011, *J.A. Wege 1874* (CANB, MEL, PERTH); 6.4 km E of Sundalara Rd on Tomkins Rd, NE of Eneabba, 30 Oct 2018, *J.A. Wege 2077* (PERTH); **South Australia**: Wanilla, some 25 km NW of Port Lincoln, 8 Nov 1968, *C.R. Alcock 2542* (AD, MEL); Granite outcrop NW of Lienerts/Woolford Track in Pinkawillinie C[onservation] P[ark], 3 Nov 2009, *T.S. Te 859 & T.S. Croft* (AD); Site BS162-MIN01101, Patchid 20131 [9.1 km direct ENE of Roger Corner, Yorke Peninsula], 13 Oct 2004, *L.M.B. Heard & N.R. Neagle BS162-1754* (AD); Verran Tanks Conservation Park, 10 Oct 1991, *D.E. Murfet 1299b* (AD); Murrunatta Conservation Park, 15 Oct 1995, *D.E. Murfet & R.L. Taplin 2270* (AD).

### 11. Levenhookia octomaculata

Taxon classificationPlantaeAsteralesStylidiaceae

F.L.Erickson & J.H.Willis, Vict. Naturalist 72: 130, figs 1–6. 1956

BD5B1111-89BA-5E9B-94D4-F25A1CA9B872

Figs 1D , 7D

#### Type.

**Australia. Western Australia**: Bolgart, 2 Nov 1953, R. Erickson *s.n.* (holotype: MEL 2295754; isotypes: K 000060079, PERTH 01025074).

#### Description.

Annual herb 3–12 cm high. Glandular hairs 0.1–0.2 mm long. Stem dark reddish brown, often paler distally, simple or branched to varying degrees with spreading to almost patent or ascending branches, somewhat sparsely glandular-hairy (especially towards the base). Leaves cauline, scattered, green or reddish brown; lamina narrowly oblanceolate to ± linear, elliptic or ovate, 2.5–15 mm long including the petiole, 0.5–2 mm wide, obtuse, sparsely glandular-hairy on the abaxial surface and margins towards the base. Flowers usually in umbels or corymbs or more rarely in short racemes, 1–ca. 150 per plant; bracts narrowly oblanceolate, lanceolate or ± linear, 2.5–11 mm long, glabrous or sparsely glandular-hairy like the leaves; pedicels 3–20 mm long, sparsely glandular-hairy. Hypanthium depressed globose or globose, 0.7–1.3 mm long, 0.7–1.5 mm wide, glandular-hairy. Calyx lobes ± equal or subequal (with the anterior pair scarcely longer than the rest), 1.2–2 mm long, acute or subacute, glabrous or sparsely glandular-hairy near the base. Corolla bright to pale pink (rarely white) with two, dark red-pink, ± elliptic markings near the base of each lobe (rarely faint or lacking?) and a creamy white throat, whitish on the reverse with mottled pink-red markings; lobes evenly arranged or with the upper (posterior) ones ± paired vertically, often weakly recurved, obovate with an attenuate base, glabrous or with a few glandular hairs abaxially near the base; anterior lobes slightly shorter and narrower than the posterior pair, 3.2–5 mm long, 1.5–3 mm wide, rounded or more rarely retuse; posterior lobes 3.5–5.2 mm long, 2–3.5 mm wide, rounded or bluntly pointed; tube creamy white, 0.2–0.4 mm long, obscured by the calyx lobes, glabrous. Labellum ventral, 2.5–3.7 mm long including a 1–2 mm long claw; hood dark red-maroon adaxially, pink abaxially and deep yellow near the cleft, sometimes prominently papillate, with a short, blunt point at the cleft apex, sparsely glandular-hairy abaxially; basal appendages yellowish or creamy white, rounded, 0.5–0.6 mm long. Column sheath white or yellowish, glabrous, 1.2–1.8 mm high on the posterior side with a thickened rim and broad anterior cleft, 3 pendulous appendages present on the inner surface of the sheath towards the throat. Column creamy white, free, erect, slightly thickened distally, 3–4 mm long; stigmatic lobes to ca. 1.4 mm long, incurved, the lowermost developing while the column is hooded, the uppermost developing later. Capsule globose or ovoid, 1.5–2 mm long excluding calyx lobes. Seeds 0.4–0.5 mm long, ca. 0.3 mm wide.

#### Diagnostic features.

*Levenhookia octomaculata* has sparsely glandular-hairy stems (especially towards the base), bracts and calyx lobes that are glabrous or sparsely glandular-hairy near the base, long pedicels (5–20 mm), a short corolla tube (obscured by the calyx lobes), and a column sheath that is usually up to half the length of the column. The corolla lobes usually have a pair of ± elliptic markings near the base and may be mottled markings on the undersurface (but see notes below).

#### Phenology.

Flowering in October and November; fruiting in November and December.

#### Distribution.

*Levenhookia octomaculata* is endemic to south-western Australia (Fig. 7C) where it is scattered across the Geraldton Sandplains, northern Avon Wheatbelt and northern Swan Coastal Plain bioregions, occurring from Eurardy Station in the north to the Chittering area in the south and extending into the Yalgoo bioregion at Mt Gibson Station.

#### Habitat.

This species grows in sand or sandy loam, usually over limestone or sandstone but sometimes in association with granite outcropping, lateritic gravel or banded ironstone. Associated vegetation is varied and includes heath, mallee heath, *Acacia* and *Calothamnus* low shrubland, *Allocasuarina campestris* shrubland, *Melaleuca* scrub, and open woodland with *Eucalyptus loxophleba* or *Melaleuca preissiana*. It can grow in sympatry with *L. stipitata*.

#### Conservation status.

*Levenhookia octomaculata* occurs in several nature reserves and national parks where it can be locally abundant. It is not currently considered to be at risk (IUCN (2012): Least Concern).

#### Etymology.

From the Latin *octo*- (eight-) and *maculatus* (spotted): a reference to the pair of markings near the base of each corolla lobe.

#### Vernacular name.

Dotted Stylewort (Erickson and Willis 1956).

#### Notes.

*Levenhookia octomaculata* was named on account of the eight prominent markings in the throat of the flower which, at the time, were thought to be unique in the genus (Erickson and Willis 1956: 133); however, eight throat markings are present in many populations of *L. stipitata* which has led to a degree of taxonomic confusion. Moreover, is not known whether all populations of *L. octomaculata* possess eight throat markings; photographs associated with *G. Byrne 600* (PERTH 06908349) suggest that they may sometimes be faint or perhaps altogether lacking. *Levenhookia octomaculata* can be reliably separated from *L. stipitata* by its bracts, which are glabrous or sparsely glandular-hairy abaxially near the base (cf. glandular hairy on the abaxial surface and margins) and calyx lobes, which are glabrous or sparsely glandular-hairy near the base (cf. moderately to sparsely glandular-hairy). It also tends to have fewer hairs on the stem and pedicels, a shorter column sheath (up to half the length of the column cf. more than half the length of the column) and a more prominently papillate labellum hood. I have observed both species growing intermixed without hybridisation at a site in Beekeepers Nature Reserve (*J.A. Wege 2074* and *J.A. Wege 2075*), at which time *L. octomaculata* was in full flower, whereas *L. stipitata* was in late flower or had finished flowering. At this site, *L. octomaculata* had unique speckled markings on the undersurface of the corolla lobes but, having made limited field observations of this species, I am uncertain whether these are consistently present.

#### Illustrations.

R. Erickson, Triggerplants 201, Pl. 57, No. 3 and 208, Pl. 58, Nos. 15–19. 1958; B.J. Grieve & W.E. Blackall, How to know W. Austral. wildfl. 4: 766, no. 7. 1982 [column sheath not shown].

#### Selected specimens examined.

**Australia. Western Australia**: 13 km E of Mummaloo-Wye Bubba Hill, Mount Gibson Station, 21 Nov 1992, *R.J. Cranfield 8562* (PERTH); Lesueur National Park, 1.8 km N of University track on Hakea track, E of Jurien Bay, 7 Nov 2007, *A. Crawford 1495* (PERTH); Petrudor Rock Reserve, SE of Dalwallinu, 7 Nov 1999, *M. Hislop 1858* (PERTH); Koolanooka Hills, 12 Oct 2005, *R. Meissner & Y. Caruso 548* (PERTH); Murchison River near Z-Bend, Kalbarri National Park, 9 Oct 1982, *K.H. Rechinger 58462* (PERTH); 6 km W on Beekeepers Rd from Brand Hwy, Beekeepers Nature Reserve, 30 Oct 2018, *J.A. Wege 2074* (AD, CANB, MEL, PERTH); Quadrat WEST 1 on Eurardy Station, ca. 43 km N of Kalbarri turn-off on the North West Coastal Highway and N of the Murchison River, 3 Oct 2003, *Wildflower Society of WA EURA 308* (PERTH).

### 12. Levenhookia chippendalei

Taxon classificationPlantaeAsteralesStylidiaceae

F.L.Erickson & J.H.Willis, Vict. Naturalist 83(5): 107, t. 2, figs 7–10. 1966

44D89005-DE1A-517A-BFD7-B195EFA02A13

Fig. 7F

#### Type.

**Australia. Northern Territory**: 39 miles [62.8 km] S of Hooker’s Creek (and ± 230 miles [370.1 km] W of Banka Banka), 12 Jul 1956, *G. Chippendale s.n.* (holotype: DNA-A0002260; isotypes: CANB 55765, MEL 2295751, PERTH 01639994).

#### Description.

Annual herb 4–35 cm high, usually with a well-developed tap root. Glandular hairs 0.1–0.3 mm long. Stem pale, green or reddish brown, much-branched near the base (rarely simple), with spreading or ascending branches, glandular-hairy. Leaves basally clustered and cauline, pale green; lamina oblanceolate or lanceolate, often narrowly so, 5–30 mm long including the petiole, 0.7–5 mm wide, acute to subacute, glandular-hairy abaxially and on the margins and sometimes on the adaxial surface towards the base. Flowers in racemes, sometimes in umbels or corymbs, 5–500^+^ per plant; bracts lanceolate to linear, 1.8–25 mm long, glandular-hairy like the leaves; pedicels 5–30 mm long, sparsely glandular-hairy. Hypanthium depressed globose or globose, 0.6–2 mm long, 0.6–2.5 mm wide, glandular-hairy. Calyx lobes equal or subequal (with the anterior pair scarcely longer than the rest), 1.2–2.5 mm long, acute, sparsely to moderately glandular-hairy. Corolla pink with a dark pink midvein and a white or yellow throat; lobes ± evenly arranged, slightly recurved, obovate with an attenuate base, rounded or scarcely apiculate, glabrous or sparsely glandular-hairy on the abaxial surface along the midvein; anterior (lower) lobes slightly shorter and narrower than the posterior pair, 3–6.5 mm long, 1.9–3.2 mm wide; posterior (upper) lobes 3.5–7.5 mm long, 2–3.8 mm wide; tube white, 0.5–1.5 mm long, shorter than the calyx lobes, glabrous or with a few glandular hairs distally. Labellum ventral, 4–6.5 mm long including a 1–1.5 mm long claw; hood pink with purplish maroon markings, sparsely glandular-hairy abaxially, papillae absent; appendage at the cleft apex pink with a yellow or white base, 1.8–3.5 mm long, incised or emarginate, glabrous; basal appendages oblong-clavate, 0.8–1.2 mm long, revolute distally, white with a yellow, papillate tip. Column sheath white, glabrous, 0.5–0.7 mm high with a thickened rim on the posterior side and 3 pendulous appendages on the inner surface towards the throat. Column whitish tipped pale pink, free, slender, 2.5–3.8 mm long, glabrous; stigmatic lobes to 1.5 mm long, incurved, the lowermost developing while the column is hooded, the uppermost developing later. Capsule depressed globose to globose or ovoid, 1.5–3 mm long excluding calyx lobes. Seeds 0.5–0.8 mm long, 0.3–0.4 mm wide.

#### Diagnostic features.

*Levenhookia chippendalei* has a glandular-hairy stem that is usually much-branched at the base, leaves that are clustered at the base and scattered along the stems, long pedicels (5–30 mm), a short corolla tube (obscured by the calyx lobes), and a prominent, incised or emarginate appendage at the tip of the labellum. A well-developed tap root is usually evident.

#### Phenology.

Flowering and fruiting from May to October, depending on seasonal conditions.

#### Distribution.

*Levenhookia chippendalei* is widespread in the arid zone in Western Australia and the Northern Territory (Fig. 7E), occurring from near Meekatharra to the Dulcie Range, north-east of Alice Springs.

#### Habitat.

*Levenhookia chippendalei* grows on sandplains or sand dunes or near salt lakes and seasonal swamps, in sand or sandy clay or in gravelly soils near waterholes or creeklines. Associated vegetation includes *Acacia* shrubland or woodland, spinifex grassland, open low scrub with *Aluta maisonneuvei* and scattered emergent *Brachychiton gregorii*, *Acacia aneura* and *Eucalyptus gamophylla*, and *Grevillea integrifolia* or *Melaleuca* tall shrubland. It may grow in sympatry with *Stylidium desertorum* Carlquist, which has similarly bright pink flowers.

#### Conservation status.

A widespread species that is not considered to be under threat (IUCN (2012): Least Concern).

#### Etymology.

Honours George Chippendale (1921–2010), who made the earliest collection of this taxon while based in Alice Springs as the Northern Territory’s first resident taxonomist.

#### Vernacular name.

Arid Zone Stylewort.

#### Illustrations.

K.F. Kenneally & A.S. George in J. Jessop, Fl. Central Austral. 363, fig. 463. 1981.

#### Selected specimens examined.

**Australia. Western Australia**: 2.2 km S of Scorpion Bore near Carnegie Homestead, 8 Sep 1973, *R.J. Chinnock 891* (AD, PERTH); 1 km S of Mount Brophy Springs, Gardner Range, 190 km SE of Halls Creek, SE Kimberley, 4 Jul 1995, *K. Coate 372* (BRI, DNA, PERTH); 20.6 km N along Canning Stock Route from Well 14, 18 Aug 2007, *R. Davis 11181* (PERTH); ca. 6.6 km on a bearing of 174 degrees from Mt Methwin, Birriliburu Indigenous Protected Area, 13 Aug 2012, *N. Gibson 6559*, *S. van Leeuwen*, *M.A. Langley & K. Brown* (PERTH); S side of Lake Kerrylyn, ca. 6.7 km on a bearing of 49 degrees from Mt Methwin, Birriliburu Indigenous Protected Area, 14 Aug 2012, *N. Gibson 6560*, *S. van Leeuwen*, *M.A. Langley & K. Brown* (PERTH); 102 miles [164 km] from Billuna, Jul 1972, *C.H. Gittins 2421* (CANB); 29 km SSE of Mount Keith, Wanjarri Nature Reserve, 29 Sep 1992, *G.J. Keighery 13011* (PERTH); Site LGS 1, 31.5 km on main track from Lorna Glen Homestead to Wiluna – Granite Peaks Rd, 10 Sep 2003, *K.F. Kenneally & D.J. Edinger K 12671 E 3868* (CANB, PERTH, MEL); 72 km NE Kiwirrburra [Kiwirrkurra], SW Lake Mackay, 21 Oct 2000, *P.K. Latz 17005* (PERTH); Little Sandy Desert, 11.4 km SW of Cooma Well, 15 Aug 1997, *S. van Leeuwen 3228* (AD, BRI, CANB, DNA, MEL, NSW); **Northern Territory**: Attack Creek, Stuart Highway, Barkley Tableland, 1 Jul 1974, *A. Beauglehole 46305* (DNA); Central Mt Stuart, 1 Jul 1974, *T. Henshall 475* (AD, DNA); Neutral Junction, 4 Jul 1974, *T. Henshall 525* (DNA); Macdonald Downs Station, 23 Oct 1974, *P. Latz 5779* (DNA); Singleton Station, 26 May 1975, *P. Latz 5962* (DNA); Tanami Sanctuary, 27 May 1976, *P. Latz 6505* (DNA, MEL); 5 km NNE of Mt Frederick, NW Tanami Desert, *P. Latz 8601*, 2 Apr 1981 (DNA).

## Supplementary Material

XML Treatment for Levenhookia

XML Treatment for Levenhookia pusilla

XML Treatment for Levenhookia murfetii

XML Treatment for Levenhookia sonderi

XML Treatment for Levenhookia dubia

XML Treatment for Levenhookia leptantha

XML Treatment for Levenhookia pulcherrima

XML Treatment for Levenhookia pauciflora

XML Treatment for Levenhookia preissii

XML Treatment for Levenhookia aestiva

XML Treatment for Levenhookia stipitata

XML Treatment for Levenhookia octomaculata

XML Treatment for Levenhookia chippendalei
